# Haematology, serum biochemistry, innate and adaptive immune system responses to blood pathogen infections in cattle: A review

**DOI:** 10.1007/s00436-026-08652-y

**Published:** 2026-04-16

**Authors:** Onyinyechukwu Ada Agina, Felix Atawal Andong, Jacinta Ngozi Omeke, Ukamaka Uchenna Eze, Madubuike Umunna Anyanwu, I. F. Jaja, James Wabwire Oguttu

**Affiliations:** 1 Department of Veterinary Pathology, Faculty of Veterinary Medicine, University of Nigeria, Nsukka, 41001 Enugu State Nigeria; 2 Department of Zoology and Environmental Biology, Faculty of Biological Sciences, University of Nigeria, Nsukka, 41001 Enugu State Nigeria; 3 Department of Veterinary Medicine, Faculty of Veterinary Medicine, University of Nigeria, Nsukka, 41001 Enugu State Nigeria; 4 Department of Veterinary Microbiology and Immunology, Faculty of Veterinary Medicine, University of Nigeria, Nsukka, 41001 Enugu State Nigeria; 5 Department of Livestock and Pasture Science, University of Fort Hare, SAMRC Microbial, Alice, 5700 South Africa; 6 Water Quality Monitoring Centre, University of Fort Hare, Alice, 5700 South Africa; 7 Department of Agriculture and Animal Health, University of South Africa, Roodepoort, Johannesburg 1710 South Africa

**Keywords:** Blood, Pathogens, Anaemia, Cattle, Serum biochemistry, Innate immune response, Adaptive immune response

## Abstract

Infection with blood pathogens, such as *Anaplasma*, Haemotropic *Mycoplasma*, and trypanosomes, has affected the livestock industry, particularly in developing countries and the tropics. Clinical signs associated with infections caused by these blood pathogens include anaemia of the haemolytic type (immune-mediated haemolytic anaemia), decreased milk yield, abortion, anorexia, cachexia, disseminated intravascular coagulopathies, conjunctivitis, ecchymoses, thrombocytopenia, leukocytosis, and death in severe cases. Immune-mediated haemolytic anaemia is more evident in anaplasmosis as the degree of anaemia is often out of proportion to the prevailing bacteriemia. Additionally, icterus, increases in serum liver enzymes and variable white blood cell count changes are observed in affected animals. Recovered cattle may become asymptomatic carriers as some apicomplexan species tend to form sequestration in the cerebrum. Asymptomatic cattle are sources of blood pathogens to competent haematophagous arthropods and consequent transfer of infection to susceptible/naive animals. Nonetheless, persistent blood infections are typically attributable to pathogens that have developed multiple strategies, including immune subversion and the dysregulation of both natural and acquired immune responses, enabling them to persist within an immunocompetent host. Therefore, this review has provided veterinary clinicians, researchers, and laboratory technicians with an overview of the haematological and serum biochemistry profiles of cattle infected with blood pathogens, prevention and control strategies, and specific innate and adaptive immune system responses, as well as the adaptive and survival properties of these blood pathogen infections in cattle.

**Graphical Abstract:**

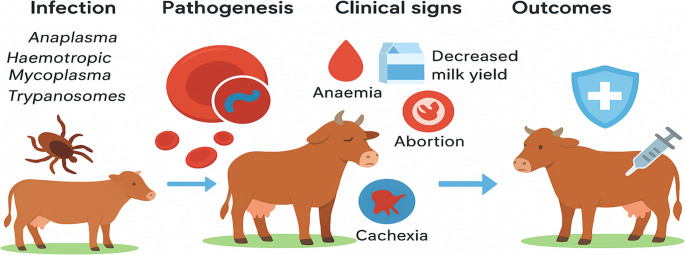

## Background

### Vector-borne pathogens of cattle

Blood-borne pathogens are transmitted through vectors such as ticks or haematophagous biting flies and are responsible for diseases of significant economic impact. These diseases can be zoonotic or non-zoonotic. The tick-borne pathogens include the *Anaplasma* spp., *Babesia* spp., *Ehrlichia* spp., haemotropic *Mycoplasma*s (haemoplasma) and *Theileria* spp. *Trypanosoma species* are transmitted by tsetse fly except for *T*. *evansi* and *T. equiperdium*, which are mechanically transmitted by blood-sucking flies such as *Tabanus* spp and sexually transmitted, respectively. These pathogens are capable of entering erythrocytes and may be found at the periphery, in the centre, or attached to the surface of the erythrocytes, as observed with haemotropic *Mycoplasma* species. Blood pathogens such as *Anaplasma* have been reported to infect neutrophils, monocytes and macrophages. Anaplasmosis and theileriosis (East Coast fever or tropical theileriosis) are commonly observed in cattle and are primarily associated with either extravascular or intravascular haemolytic anaemia. (Ashuma et al. 2013; Jalali et al. 2018; Lawrence et al. 2018). Disease severity depends on host immunity, nutrition, age, breed, gender, and pathogen virulence. Blood pathogen infections can cause fever, jaundice, anorexia, lethargy, and nervous disorders in severe cases. Animals that recover often become asymptomatic carriers within the herd. Control of these diseases is usually targeted towards early diagnosis and treatment, with regular screening of the animals to control the disease and the use of tick control programmes, chemoprophylaxis, chemotherapy and vaccination (Sánchez et al. 2015; Almazan et al. 2018).

### Anaemia as a major clinical sign of blood pathogen infections in cattle

Anaemia can be defined as a decrease below the reference range of packed cell volume or haematocrit, haemoglobin concentration and/or red blood cell count (Stockham and Scott 2008; Roland et al. 2014). However, these parameters may not be uniformly decreased because of the presence of abnormal erythrocytes with an abnormal erythrocyte volume or intracellular haemoglobin concentrations. Clinical signs associated with anaemia reflect decreased oxygen carrying capacity to tissues which include weakness, pallor of mucous membrane of the gingiva, conjunctiva and vulva, loss of stamina, rapid respiration, increased sensitivity to cold, tachycardia, hypoxia, weak pulse and shock (Brockus 2011). Anaemia is classified based on bone marrow responsiveness and can also be classified according to the erythrocyte morphology (cell size and haemoglobin content), and pathophysiology (aetiological classification). In bone marrow responsiveness, anaemia can be categorised into regenerative or non-regenerative anaemia and is primarily based on the presence or absence of reticulocytosis in blood (Brockus 2011; Katsogiannou et al. 2018). Reticulocytosis leads to a progressive increase in haematocrit, and this is termed effective erythropoiesis. Therefore, in this case, anaemia is said to be regenerative. Reticulocytosis is typically established by determining the corrected reticulocyte percentage (CRP) or reticulocyte production index (RPI), along with increased polychromasia. In other words, a corrected reticulocyte percentage of greater than 1% in cattle is considered an indication of a regenerative response (Cowell 2004). Cattle have a mild ability to produce reticulocytosis when compared to other mammalian species (Roland et al. 2014). Normoblastaemia may be present in anaemic animals, and erythrocytes with basophilic stippling in haemolytic anaemias frequently accompany increased polychromasia. Macrocytic hypochromic indices, anisocytosis, Howell-Jolly bodies, rubricytosis, codocytosis or basophilic stippling are erythrocytic abnormalities that support a regenerative status. A mild to moderate erythroid hyperplasia with or without reticulocytosis denotes a regenerative anaemia (Allison and Meinkoth 2010).

In cattle, pathogens infecting ruminant RBCs include protozoa (*Babesia* spp., *Theileria* spp., *Trypanosoma* spp. and *Sarcocystis* spp.), and bacteria (*Anaplasma marginale*, *Mycoplasma wenyonii*,* Leptospira* spp., and *Clostridium* spp.). Babesia spp. are large, penetrate the red cell, then multiply by binary fission and ultimately rupture the red cell, causing intravascular haemolysis. Severe intravascular haemolytic anaemia in acute bovine babesiosis is characterised by haemosideuria, haemoglobinuria, and haemoglobinaemia. Mild forms of haemolytic anaemia in babesiosis may or may not produce haemoglobinaemia because, in mild cases, the small amount of circulating free haemoglobin binds to haptoglobin and is removed by the healthy liver, such that haemoglobinuria does not occur. Extravascular haemolytic anaemia involves the destruction and lysis of red blood cells engulfed by macrophages. Basophilic stippling, which appears as blue dots in the RBC cytoplasm, occurs in regenerative anaemias in ruminants.

### Immune-mediated haemolytic anaemia in blood pathogen infections

Immune-mediated haemolytic anaemia (IMHA), also known as autoimmune haemolytic anaemia, occurs as the destruction of red blood cells with surface-bound immunoglobulins or erythrocyte precursors. The majority of autoimmune haemolytic anaemia cases are attributable to underlying infectious diseases, neoplastic disorders, or administration of certain drugs. It has been reported that *Anaplasma marginale* and *Theileria* spp. can cause IMHA (Nazifi et al. 2008). Spherocytes are evident in blood smears of cattle with immune-mediated haemolytic anaemia, though their recognition is difficult because normal cattle erythrocytes are small (Nassiri et al. 2011). While the exact pathophysiological mechanism of IMHA in anaplasmosis remains unclear, it is believed that these intraerythrocytic blood pathogens modify the erythrocyte membrane, leading to a quick removal of abnormal red blood cell shapes from the peripheral circulation. Translocation of phosphatidylserine molecules from the inner leaflet of the cell membrane to the external surface of RBCs, especially in bovine theileriosis, can induce an antibody response for phagocytic clearance of such RBCS by macrophages (Shiono et al. 2003). In anaplasmosis, anaemia occurs from the extracellular destruction of infected red blood cells. The intensity of anaemia in acute anaplasmosis is blown out of proportion as the host produces antibodies against both infected and non-infected red blood cells. In other words, there is immune-mediated destruction of both the blood pathogen and host cells. Both extravascular and intravascular haemolysis, which occur in regenerative IMHA, are usually associated with a strong regenerative response (Jalali et al. 2018).

Haemolysis of erythrocytes occurs following attachment of the trypanosomes onto the red cell surface via sialic acid receptors. Anaemia occurs by erythrophagocytosis of macrophages in the liver, spleen and bone marrow, haemosiderosis in macrophages in some organs, reduced cell survival and occasional haemoglobineamia. Hypersplenism in trypanosomosis is caused by an exaggerated function of the spleen and resultant marked increase in cellularity of the spleen, which impedes blood cell movement and delays the red cells longer than normal in the red pulp of the spleen. This, however, causes damage to the red cells due to low pH and oxygen tension. In this review, it should be noted that the term ‘trypanosomosis’ is used interchangeably with ‘trypanosomiasis’. Trypanosomosis is commonly preferred because of its common usage in veterinary and animal health contexts. Hypersplenism and haemodilution (increased serum volume) are evident in trypanosomosis and cause relative anaemia. Dyserythropoiesis in trypanosomosis has been attributed to the destruction of normoblasts and reticulocytes by marrow macrophages. Bone marrow hypoplasia observed in ruminant trypanosomosis has been attributed to low utilisation of iron due to storage of iron by macrophages in the spleen (Stijlemans et al. 2015, 2018). Additional factors contributing to anaemia in trypanosomosis are mechanical damage to erythrocytes caused by the whipping motion of trypanosome flagella, fever (pyrexia), platelet clumping, as well as the presence of toxins and metabolites produced by the trypanosomes. (proteases, neuraminidase, phospholipases, free fatty acids), lipid peroxidation and malnutrition. Tumour necrosis factors have been incriminated in dyserythropoiesis. Mechanical damage to endothelial cells occurs during tissue invasion by trypanosomes, for example, in cases of *T. brucei* infection. Undulating fever increases the osmotic fragility of erythrocytes and enhances the permeability of the erythrocyte membrane. Thermal induction increases lipid peroxidation of erythrocytes. Proteases and other active chemicals are released by both active and lifeless trypanosomes, and they can cause erythrocyte damage. The production of superoxide and free radicals after lipid peroxidation, which results in oxidative haemolysis, is closely linked to the cause of anaemia in trypanosomosis. The development of antigen-antibody complexes with sialic acid may be linked to anaemia in trypanosomosis, which can happen during erythrophagocytosis. (reviewed in Mbaya et al. 2012). Haematopoietic cytokines, including tumour necrosis factor-alpha (TNF-α), are associated with anaemia in trypanosomosis because increased levels of TNF-α can reduce erythropoietin production and effectiveness.

Haemoplasms such as *Mycoplasma wenyonii* (formerly *Haemobartonella wenyonii*) are epi-erythrocytic pathogens that attach onto the surface of red blood cells. The affected red cells rupture when the host produces antibodies against the bacterial antigens, which are attached to the red cell surface or antigens exposed on altered membranes. Macrophages in the spleen, liver, and haemolymph node engulf and lyse coated red blood cells, causing haemolytic anaemia. The anaemia observed in this condition is the anaemia of the macrocytic hypochromic type without haemoglobinaemia. Anaemia is usually moderate to severe, with an increased number of reticulocytes and polychromasia (Allison and Meinkoth 2010).

## Selected blood pathogens of cattle

### *Anaplasma* species

The genus *Anaplasma* belongs to the family *Anaplasmataceae* within the order *Rickettsiales* of class Alphaproteobacteria. *Anaplasmataceae* are classified based on similarities in 16 S ribosomal RNA gene (16 S rDNA or *rrs*), or in combination with 60 kDa heat shock protein gene (*groESL*), beta subunit of RNA polymerase (*rpoB*), citrate synthase (*gltA*) and major surface protein (MSP) (Dumler 2011; Ybañez et al. 2014; Kolo et al. 2020; Junsiri et al. 2020). The genetic diversity of the family *Anaplasmataceae* (*Anaplasma*,* Ehrlichia*,* Neorickettsia* and *Wolbachia*) has been completely studied with the help of molecular markers (Kocan et al. 2015; Battilani et al. 2017), and encompasses several species of veterinary importance (Llanes and Rajeev 2020). The members of this family can reside within a cytoplasmic vacuole as inclusion bodies (mulberry-like bacterial clumps called morulae) in mammalian cells such as endothelial cells, erythrocytes, and x (Dumler et al. 2001). They are small, coccoid, pleomorphic or ellipsoidal, and the size in diameter ranges from 0.3 to 0.4 microns (Llanes and Rajeev 2020). Anaplasmosis constrains cattle production due to low weight gain, low milk yield, abortion, cost of treatment and mortality (Kocan et al. 2015). Thus, it is one of the major causes of economic loss, especially in developing countries where it is endemic (Rodríguez et al. 2009; Peter et al. 2020). The huge economic loss caused by bovine anaplasmosis in many parts of the world is estimated to be the tune of USD 300–800 million (Kocan et al. 2003). About USD 875 million loss has been attributed to disease burden and control of anaplasmosis in South America (reviewed in Brown 1997) and USD 30.5 million in Australia (Bock and de Vos 2001). The *Anaplasma* spp. are the *Anaplasma marginale*, *A. bovis*,* A. ovis*,* A. capra*,* A. phagocytophium*,* A. platys*, and *A. centrale* that affect ruminants (cattle, buffalo, sheep, goat, reindeer), and non-ruminants (human, dog) may act as carriers (Palomar et al. 2015; Rjeibi et al. 2017; Chien et al. 2019). Both *A. platys* and *A. phagocytophilium* were formerly under the genus *Ehrlichia* but were properly reclassified as *Anaplasma* (Dumler et al. 2001). A novel species of *Anaplasma*,* Candidatus Anaplasma camelii*, was first reported by Bastos et al. (2015), and this pathogen has been associated with human and animal (dromedary camel and cattle) infections. It is transmitted by *Hyalomma asiaticum* ticks in China (Kang et al. 2014). This new species is closely related to *Anaplasma platys* and *Ehrlichia canis*, and has been detected in cattle and deer in Malaysia (Koh et al. 2018). *Candidatus Anaplasma boleense*, a novel species, has also been detected in Malaysian wildlife (wild boars, bats and squirrels) (Koh et al. 2016).

Phylogenetic analyses showed that two clades exist within the genus *Anaplasma*: erythrocytic (*A. marginale*,* A. ovis and A. centrale*) and leukocytic (*A. bovis*,* A. phagocytophilum*, and *A. platys*) (Ybañez et al. 2014), and these are clinically relevant *Anaplasma* species (Iqbal et al. 2019). *Anaplasma marginale* is an obligate, intracellular, gram-negative bacterium that resides in host cell cytoplasm enclosed in a host cell-derived membrane-bound vacuole (Rodríguez et al. 2009). The bacterium is capable of infecting endothelial cells in vivo, and these cells may serve as a reservoir for infection (Carreño et al. 2007; Wamsley et al. 2011; Guarnizo et al. 2020). It causes bovine anaplasmosis (gall sickness), a debilitating and potentially fatal tick-borne disease of cattle (Shebish et al. 2012; Peter et al. 2020). Ticks such as *Dermacentor* and *Rhipicephalus* can biologically transmit anaplasmosis (Jaswal et al. 2015). Mechanical transmission by biting flies such as *Stomoxys calcitrans*,* Haematobia irritans* and *Tabanus* species has been reported (Ashuma et al. 2013), and by blood-contaminated fomites such as surgical, dehorning, castration, tattoo instruments and hypodermic needles (Ashuma et al. 2013). In addition to mechanical and biological transmission, *A. marginale* may also be transmitted from cow to calf transplacentally (Kocan et al. 2003). The disease affects tropical and sub-tropical parts of the world, such as Africa, the Middle East, Asia, Australia, Southern Europe, and Central and South America (Kocan et al. 2000) and in temperate areas (Futse et al. 2003). The severity of anaplasmosis is highly dependent on the host’s immune status and coinfections by other pathogens (Silaghi et al. 2017).

The major surface protein (MSP) is used to differentiate *A. marginale* strains, which differ in morphology, protein sequence, antigen characteristics and their ability to be transmitted by ticks (Kocan et al. 2015). Mortality caused by *A. marginale* is usually high in animals over two years of age, cattle less than 2 years old are resistant to the clinical disease, but if infected, can become chronic carriers. The reason for this could be immune protection by passively transferring maternal antibodies in the calves (Kocan et al. 2015).The major surface protein (MSP) is used to differentiate *A. marginale* strains, which differ in morphology, protein sequence, antigen characteristics, and their ability to be transmitted by ticks (Kocan et al. 2015). Mortality caused by *A. marginale* is usually high in animals over two years of age; cattle less than 2 years old are resistant to the clinical disease, but if infected, can become chronic carriers. The reason for this could be immune protection by passively transferring maternal antibodies in the calves (Kocan et al. 2015).

Bovine anaplasmosis is characterised by fever, anorexia, severe haemolytic anaemia associated with intraerythrocytic parasitism, pale mucous membrane, yellowish urine, jaundice, depression, loss of production, weight loss, abortions, tachypnoea, dehydration, constipation, incoordination, occasional aggressiveness, and a decrease in milk production and death in acute infections (Shebish et al. 2012; Ola-Fadunsin et al. 2018a, 2018b). The death of infected cattle is consequent upon severe anaemia. Cattle that recover become asymptomatic carriers characterised by cyclic and low-level parasitaemia, and if they are not treated with the proper course of antibiotics (Shebish et al. 2012). *Anaplasma centrale* infection is defined by a gradual onset of mild anaemia, accompanied by the appearance of inclusion bodies occurring within the erythrocytes (Rjeibi et al. 2017). It causes the sub-clinical form of anaplasmosis. *Anaplasma phagocytophilum* infects neutrophils and causes human granulocytic anaplasmosis. It is also known to be zoonotic (Dumler 2011; Lee et al. 2017). In humans, it can be transmitted through organ transplantation and blood transfusion (Annen et al. 2012; Jereb et al. 2012). There is yet no report of transmission of *Anaplasma* species to humans via animal products (McDaniel et al. 2014). *Anaplasma phagocytophilum* has also been detected in cattle by PCR (Iqbal et al. 2019). In cattle, the disease is characterised by pyrexia, cough, low milk yield in dairy cattle and anorexia (Woldehiwet 2008; Han et al. 2018). The bacterium infects blood cells, but there is no evidence of direct neutrophil tropism in cattle as seen in humans. (Aktas et al. 2011). *Anaplasma (Ehrlichia) platys* infects dogs, ruminants, and horses and poses a potential risk to humans. It has been associated with leukopaenia caused by neutropenia and thrombocytopaenia in affected mammals (Dyachenko et al. 2012a, b). The basophilic inclusion of *A. platys* has been demonstrated in peripheral blood platelets of dogs, causing a disease called infectious canine cyclic thrombocytopaenia (Inokuma et al. 2002). It is associated with ecchymotic haemorrhage and immune-mediated thrombocytopenia due to proliferation of the pathogen during the initial phase of the infection (Dyachenko et al. 2012a, b). A complete genome sequencing of *A. platys* showed notable differences when compared to the genomes of other *Anaplasma* species. There was an apparent absence of *rbfA*, a gene encoding a 30 S ribosome-binding factor, which acts as a cold-shock protein, and two other genes that are involved in biotin metabolism (Llanes and Rajeev 2020). There were also differences in the gene families encoding the outer membrane proteins, type IV secretion system, and ankyrin repeat-containing proteins, and these gene families possess highly divergent sequences that are associated with immune subversion and persistence in the host (Llanes and Rajeev 2020).

*Anaplasma bovis* morulae are found in monocytes and macrophages of infected cattle, and it is prevalent in Africa, Asia and South America (Dumler et al. 2001). The DNA fragments have been detected in dogs, raccoons, deer, cattle and Japanese wild cats (Jilintai et al. 2009; Sakamoto et al. 2010; Ybañez et al. 2014; Iqbal et al. 2019). *Anaplasma bovis* infection is rare and subclinical in nature (Jilintai et al. 2009). *Anaplasma centrale* is centrally located in red blood cells, has a different morphology and mild virulence when compared to *A. marginale* (Decaro et al. 2008). Although the disease caused by A. centrale can be severe, the infection can be asymptomatic or present with mild anaemia. Thus, *A. centrale* is used for vaccination *against A. marginale* infection in endemic areas.

Risk factors associated with bovine anaplasmosis include cattle breed, herd and farm sizes, farm age, production type, herd owner, presence of ticks, frequency of tick control, closeness to waste areas, closeness to bushes, closeness to water bodies, closeness of farm to human settlement and frequency of prophylactic treatment against blood pathogens (Ola-Fadunsin et al. 2018a, 2018b). In areas where the disease is prevalent, native cattle breeds have acquired a natural tolerance to tick infestations and anaplasmosis (Jonsson et al. 2008).

### Life cycle and pathogenesis

**Fig. 1:**
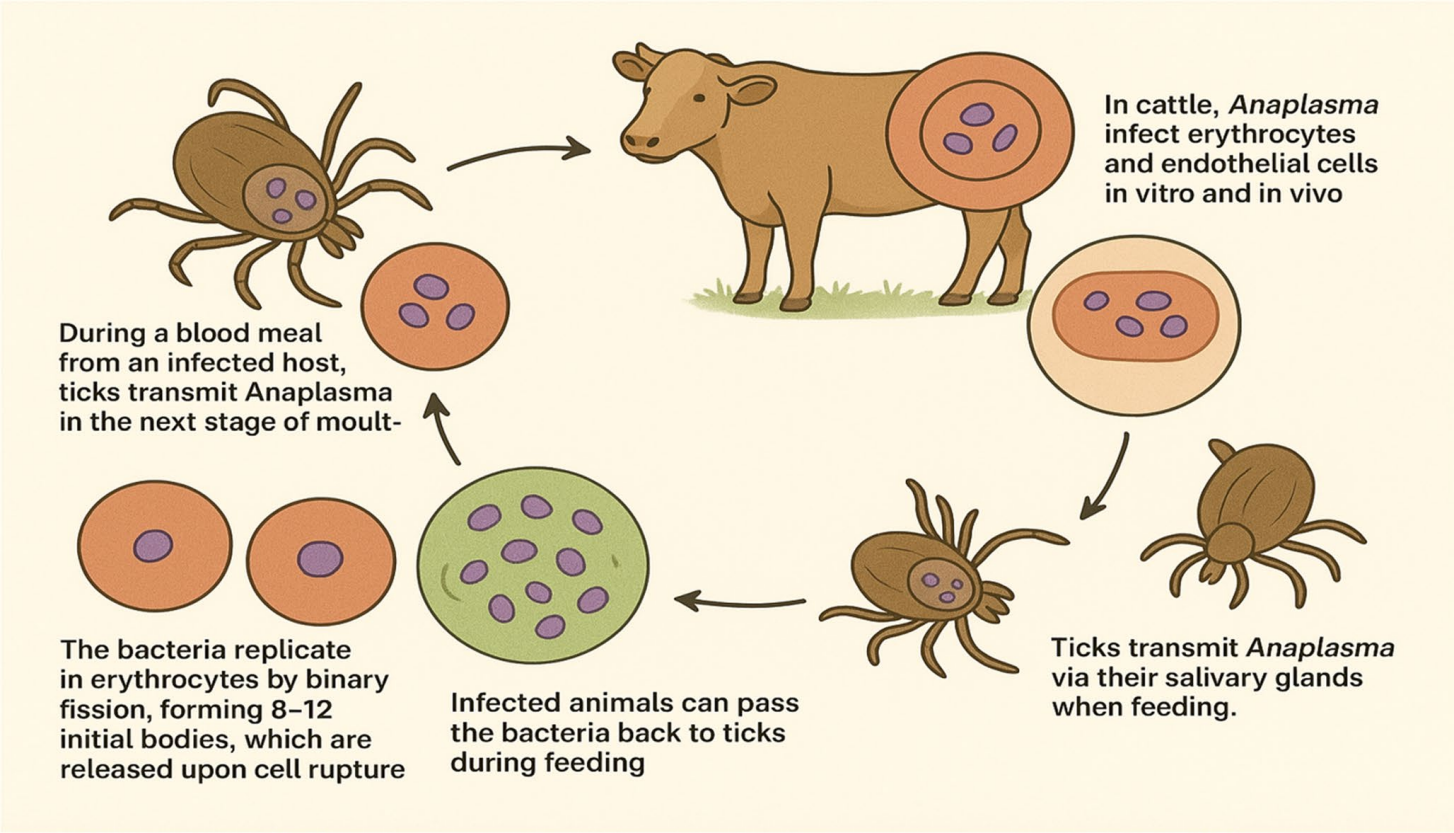
The life cycle of *Anaplasma marginale* in cattle

Members of the Family *Anaplasmataceae* have a complex life cycle that involves vertebrate hosts and invertebrate vectors (Llanes and Rajeev 2020). Thus, there is a complex interaction between *Anaplasma* and the bovine’s host cells (endothelium and erythrocytes) (Carreño et al. 2007). Ticks transmit *Anaplasma* during their subsequent moulting stage following a blood meal from an infected host. In cattle, *Anaplasma* invades erythrocytes and endothelial cells both in vitro (Munderloh et al. 2004) and in vivo (Carreño et al. 2007; Wamsley et al. 2011). Ticks transmit *Anaplasma* through their saliva during feeding. The bacteria multiply in red blood cells via binary fission, releasing 8–12 initial bodies upon cell rupture to infect more erythrocytes. Infected animals can re-infect ticks. Within ticks, the bacteria appear in gut epithelial cells, replicate in the midgut and salivary glands, and are passed to new hosts by moulted ticks after several cycles (Fig. 1) (Kocan et al. 2010). Cattle from low-endemic regions are at risk when moved to areas with high tick density and endemicity (Almazán et al. 2008).

The pathogenesis of anaplasmosis begins following either vertical or horizontal transmission to a previously unexposed host. Symptoms such as anaemia occur after a latent period due to the replication of *A. marginale* or *A. centrale* within red blood cells (Parvizi et al. 2020).

### Innate and adaptive immune responses to *Anaplasma marginale* infection

Because *Anaplasma marginale* is unable to replicate in cells that express MHC molecules, the development of protective immunity against the pathogen necessitates the coordinated activation of CD4⁺ T cells (helper T cells) and antibody-mediated responses (reviewed in Brown 2012). The Helper T cells support B cells in switching to high-affinity IgG subclasses that improve bacterial opsonization. Moreover, interferon-gamma released by CD4⁺ T cells activated in response to *Anaplasma* antigens induces macrophage production of microbicidal compounds and promotes IgG2 synthesis by B cells (Estes et al. 1994). During both the acute and chronic phases of infection, the host produces strong IgG1 and IgG2 responses that fluctuate over time, with IgG titres typically sustained between 3,000 and 100,000 in the chronic stage (Han et al. 2010). The IgG response is primarily directed against the outer membrane major surface protein 2 (MSP2), which is immunodominant and a variable antigen outer membrane protein (Lopez et al. 2005). IgG antibodies are also produced against other MSPs (MSP3, MSP1, MSP4 and MSP5) (Lopez et al. 2005). It is presumed that IgG2 antibody directed against predominant variants of MSP2 is responsible for controlling the initial acute parasitaemia, as well as subsequent peaks of parasitaemia that occur during the persistent infection due to the appearance of novel antigenic variants (French et al. 1999). The IgG antibody acts by neutralising the extracellular bacteria before they invade new erythrocytes or opsonising the bacteria for phagocytosis by macrophages. The generation of antibodies targeting new MSP2 variants does not effectively clear the infection.; therefore, the animal become a chronic carrier for life. Brown (2012) stated that antibodies directed against infected red blood cells are undetectable, resulting in the absence of phagocytosis of the infected red cells was thought to be by other mechanisms, but Jalali and co-workers (2018) believe that the host produces antibodies against parasitised and non-parasitised erythrocytes resulting in immune-mediated extravascular destruction, hence the reason for the anaemia that is blown out of proportion in bovine anaplasmosis.

*Anaplasma marginale* has an outer membrane that induces antigen-specific T cell response, which leads to rapid proliferation of T lymphocytes in the blood and spleen, and interferon production, conferring complete protection from the infection and disease (Han et al. 2010; Morse et al. 2012). The cells that responded were identified as CD4 + T cells, whereas CD8 + T cells and γδ T cells did not exhibit a response. Humoral response is characterised by high IgG1 and IgG2 antibody titres of about 30,000, following 2–3 weeks of infection. Isotype switching is dependent on the CD4 + mechanism. Antibodies directed against the MSP2 are not enough to clear the infection. Cattle immunised with *A. marginale* purified outer membrane protein induced complete protection against virulent challenge (Noh et al. 2010). The complete protection observed against the heterologous strain challenge was primarily driven by immune responses targeting conserved, subdominant antigenic epitopes within the outer membrane proteins used for immunization (Lopez et al. 2008; Sutten et al. 2010). These immunogenic proteins, e.g. VirB9-1, VirB9-2 and VirB10, expressed on the *A. marginale* outer membrane induced the strongest IgG and T helper cell responses, and they conferred protective immunity (Morse et al. 2012). Therefore, the host responses to *A. marginale* invasion are characterised by strong IgG responses and a T helper response. These outer membrane proteins are excellent vaccine candidates against bovine anaplasmosis.

The pathogenesis of bovine anaplasmosis involves antigen-presenting cells that display MHC class II molecules, which are in or near the site of tick bite, or cells that are part of the circulatory system, e.g. endothelial cells of the microvasculature (Munderloh et al. 2004). Anaemia occurs because of the phagocytosis of infected erythrocytes (extravascular haemolysis) by spleen phagocytic cells. While acute infection is typically managed, it may not be eliminated from the host; affected animals can remain persistently infected throughout their lives with variable levels of parasitaemia (Han et al. 2010). Therefore, high antigen loads are a hallmark of both acute and chronic stages of infection (Brown 2012).

### Evasion of the immune system: adaptive and survival properties

One of the characteristics of intracellular pathogens is their ability to maintain infection in the host even in the presence of innate and adaptive immunity (reviewed in Byndloss and Tsolis 2016). Persistent infections are usually asymptomatic but become risky when certain factors, such as poor nutrition or pregnancy, reactivate the dormant infection (Thakur et al. 2019). *A. marginale* outer membrane protein A (AmOmpA) enables the bacterium to invade mammalian and tick cells (Hebert et al. 2017). Persistent infections are usually caused by pathogens that have evolved several strategies to remain in an immunocompetent host. Such strategies involve subversion or dysregulation of the host’s innate and adaptive immune systems (reviewed in Sacks and Sher 2002). *Anaplasma phagocytophilium* inhibits the neutrophil killing mechanisms and prevents neutrophil apoptosis. *Anaplasma* species circumvent adaptive immunity by employing antigenic variation in their major surface proteins—MSP2/P44 in *A. phagocytophilum* and MSP2 and MSP3 in *A. marginale* (Brown 2012).

### Evasion of the innate immune response by *Anaplasma* species

*A. marginale* and *A. phagocytophilum* do not possess genes responsible for the biosynthesis of lipopolysaccharide and peptidoglycan, which are pathogen-associated molecular patterns known to activate macrophages via toll-like receptors (TLRs) and Nod-like receptors (NLRs). These PAMPS are mainly expressed by Gram-negative bacteria, but not expressed by *A. marginale* and *A. phagocytophilium* (Sorbara and Philpott 2011). *Anaplasma marginale* inhabits cells such as erythrocytes, which do not possess phagocytic function; hence, it lacks the innate defence mechanisms. *Anaplasma phagocytophilium* can reside within the hostile environment of the neutrophil because of several adaptive properties that enable it to avoid the antimicrobial function of the neutrophil (reviewed in (Rikihisa et al. 2010). It binds to cell surface glycans, bypassing complement and Fc receptor-mediated phagocytosis. It blocks superoxide anion production by scavenging free radicals and inhibiting NADPH oxidase. It also induces neutrophil IL-8 secretion, recruiting more neutrophils that can invade and facilitate bacterial replication within erythrocytes. (Carlyon and Fikrig 2006). In sheep, *A. phagocytophilium* can evade and suppress the innate immune system by causing severe leukopenia associated with lymphopenia, prolonged neutropenia and thrombocytopaenia at the early phase of the infection, which increases the animal’s susceptibility to secondary bacterial infections (Whist et al. 2002; Borjesson et al. 2005). There is a downregulation of IL-2 receptor CD25 in activated ovine CD4 + T cells andT regulatory cells, thereby causing impairment of 2 subsets of T lymphocytes (Woldehiwet 2008).

*Anaplasma marginale and A. phagocytophilium* possess a type IV secretion system (T4SS). This system secretes virulence-effector proteins that facilitate the binding to and entry into erythrocytes and tick cells (reviewed in Gillespie et al. 2010; Rikihisa et al. 2010). The T4SS effector proteins, like ankyrin repeat domain-containing protein A (AnkA) are expressed in host erythrocytes (Brown 2012). Their immunogenicity, strain conservation, and surface localisation make them strong vaccine candidates. (Morse et al. 2012; Quiroz-Castañeda et al. 2016a, 2016b).

### Evasion of the adaptive immune response

*Anaplasma* sustains its persistence in nature and transmission to new hosts by employing antigenic variation in the highly immunogenic and immunodominant MSP2 protein and the closely related MSP3 protein, which possesses a shared C-terminal region with MSP2 (Rodríguez et al. 2009). The MSP2 locus of *A. marginale* contains a variable number of non-functional segments of DNA that resemble functional genes. The incorporation of genetic material from pseudogenes, either in its entirety or as discrete segments, into the MSP2 expression site through gene conversion underlies the mechanism of antigenic variation for MSP2 and MSP3. This process, however, produces multiple variants that are observed during persistent infection. (Brayton et al. 2003; Meeus et al. 2003). Six major membrane surface proteins (Msp1α, Msp1β, Msp2, Msp3, Msp4, and Msp5) of *A. marginale* have been extensively investigated (Rodríguez et al. 2009), but MSP2 is the dominant protein recognised by sera from *A. marginale-*infected cattle or cattle immunised by surface protein. The MSP2-specific antibody response is predominantly directed towards the hypervariable region and not the conserved region, which is rich in T and B lymphocyte epitopes (Brown et al. 2003; Abbott et al. 2005). The hypervariable region is exposed on the surface (Zhuang et al. 2007), and the phenomenon of antigenic variation becomes apparent under conditions where immune selection is operative (Abbott et al. 2004). Emerging *A. marginale* variants have been shown to evade anti-MSP2 antibodies during persistent infection. (Brown 2012). Failure to prevent *A. marginale* infection is attributable to the emergence of other MSP2 variants (IJdo et al. 2002). Thus, by utilizing segmental gene conversion to vary its major surface protein MSP2, *A. marginale* effectively avoids opsonization and neutralization by host antibodies, allowing it to escape adaptive immunity. (Meeus et al. 2003).

In addition, Han et al. (2008) reported that *A. marginale* evades the immune system by quickly eliminating antigen-specific CD4 + T cells and suppressing interferon production during infection. The resulting absence of immunologic memory in cattle challenges the idea that established infections prompt rapid memory CD4 + T cell responses upon re-infection. (Abbott et al. 2005). Nothing is known about what is responsible for the deletion of a physiologically relevant number of antigen-specific CD4 + cells following infection with *A. marginale* (Han et al. 2008). Possible suggestions include high antigen load in persistent infection, which drove the antigen-specific-CD4 + cells to physical deletion by activation of induced cell death, sequestration in the spleen, or antigen-specific-CD4 + cells became anergic or antagonistic from exposure to antigenically variant epitopes (Abbott et al. 2005; Han et al. 2008).

### Haematology and serum biochemistry

*Anaplasma marginale* infection in cattle is characterised by progressive and marked anaemia (a significant decrease in RBC count, haemoglobin and PCV levels) (Coşkun et al. 2012; Ashuma et al. 2013). The serum biochemical profile shows elevated levels of serum total protein and total bilirubin, as well as increased activities of alanine aminotransferase (ALT) and aspartate aminotransferase (AST). Anaemia, hyperbilirubinaemia and increased serum liver enzyme activities were indicative of hepatic insufficiency or hepatocellular damage (Coşkun et al. 2012). Also, due to hepatic insufficiency, conjugation of a massive amount of bilirubin released during excessive destruction of erythrocytes is reduced, hence the increase in free bilirubin in serum (icterus). Increased serum globulin concentration indicates an increase in immunoglobulin concentration, confirming the host’s immune response to counter the infection (Ashuma et al. 2013). Cattle with low parasitaemia exhibited normal total leukocyte counts, whereas leukocytosis was observed during the incubation period and progression to the clinical stage of the disease. A normal leukocyte count suggests subclinical or chronicity in the affected cattle. *Anaplasma bovis* infection has been observed to cause anaemia of varying severity, mild leukocytosis, and thrombocytopaenia in cases where there is co-infection with other *Anaplasma* species and *Theileria* species (Ooshiro et al. 2008). *Anaplasma marginale* infection in Arabian one-humped camels is characterised by a significant decrease in RBC count, Hb and PCV, increased mean corpuscular volume (MCV) and erythrocyte sedimentation rate (ESR). Thus, a macrocytic normochromic anaemia was observed in the affected camels. Leukocytosis was observed, characterized by an increased lymphocyte count and accompanying neutropenia. Serum biochemical profile of the *A. marginale* infected camels includes an increase in serum activities of AST and ALT, hyperbilirubinaemia, increased blood urea nitrogen and high icteric index with hypoproteinaemia (Alsaad 2009). *Anaplasma marginale* and bovine viral diarrhoea virus co-infection in Holstein-Friesian cows caused substantial decreases in RBC, haematocrit, Hb concentration and glucose level. Mild hypoalbuminaemia with moderate to increased serum activities in AST, ALT, GGT, ALP, CK and LDH, high serum total and direct bilirubin levels were observed (Szabara et al. 2016). Experimental infection with *A. marginale* in splenectomised calves and buffaloes is characterised by a severe decrease in red blood cells, haemoglobin, PCV and platelets. Both splenectomised and non-splenectomised cattle and buffaloes had shown an increase in MCV and leukocyte count due to elevation in lymphocyte numbers (Lima et al. 2019).

### Diagnosis of *Anaplasma* species infection

Tentative diagnosis is based on clinical signs such as increased meat and milk production losses, cachexia, abortion, icterus, and death. Confirmatory diagnosis is usually based on microscopic examination of Giemsa-stained blood smears, serological tests, and molecular diagnosis. In cattle showing the clinical or acute form of the disease, examination of thin blood smears by light microscopy has been performed routinely to detect *Anaplasma* morulae at the margin or centre of the red blood cell cytoplasm, but it is less sensitive in determining asymptomatic, pre-symptomatic and carrier animals (Ashuma et al. 2013). Serological testing involves measurement of *Anaplasma-*specific antibodies by employing the complement fixation test, card agglutination test, and indirect immunofluorescent antibody test (Salm et al. 2011), enzyme-linked immunosorbent assay (ELISA) (Parvizi et al. 2020) and recombinant MSP5-based competitive-ELISA, which is highly sensitive in detecting subclinical infection (Strik et al. 2007; El-Naga et al. 2009). Competitive ELISA (cELISA) test is highly recommended for monitoring and screening of cattle populations with *A. marginale* and *A. centrale*. Because of cross-reaction with other *Anaplasmataceae* such as *A. ovis*, *Ehrlichia Chaffeensis* and *A. phagocytophilum*, it is difficult to differentiate the *Anaplasma* species; therefore, the use of highly sensitive and specific molecular techniques, such as reverse line blot hybridisation assay, conventional and quantitative real-time PCR, can differentiate the *Anaplasma* species even in animals with low parasitaemia (Carelli et al. 2007; Hove et al. 2018; Ola-Fadunsin et al. 2018a, 2018b). PCR-restriction fragment length polymorphism has been employed in *A. marginale* detection in Pakistani cattle (Ashraf et al. 2013). The use of PCR discriminates *A. marginale* from the closely related species *A. centrale*, which is used for vaccination. Vaccination with *A. centrale* has been deemed to be cost-effective for many countries, despite the risk of transmitting emerging pathogens along with the vaccine (Potgieter & Stoltsz 2004). The variable region of the MSP1α gene is used for differentiating the isolates. The high level of variation in *A. marginale* outer membrane proteins (OMPs) makes them potential vaccine candidates (Hove et al. 2018).

### Prevention and control strategies

A good understanding of the epidemiology of bovine anaplasmosis in a certain geographical area is helpful in the monitoring and control of this disease. Prevention and control are based on regular monitoring, timely treatment (chemotherapy and chemoprophylaxis; oxytetracycline, chlortetracycline, imidocarb, doxycycline and rifampin) and countermeasures against ticks, such as the use of acaricide, and vaccination against tick infestation and tick-borne pathogens (Kocan et al. 2003). Tick vaccines prevent and protect against tick feeding and reproduction, but are not yet available for field use (Guerrero et al. 2012). Acaricide-resistant ticks and the detection of acaricide residues in meat and milk limit the use of acaricides in food animals. Chemoprophylaxis is effective but only for a short period. Vaccination with live or attenuated vaccines is the method of choice against anaplasmosis by using *A. centrale.* The strain is less virulent, safe, protects against the clinical signs and provides cross-protection to several field and virulent isolates (Rodríguez et al. 2009) and it is used in many parts of the world such as Africa, South America, Israel and Australia (Bock and de Vos 2001; Bell-Sakyi et al. 2015), but immunity may be short-lasting, and if infection is not prevented, the animals may become carriers for life (Coetzee et al. 2006). The advantage of the use of live vaccine is that there is a risk of co-transmission of other ruminant pathogens and newly emergent pathogens, and haemolytic diseases in calves born to immunised cows (Dark et al. 2011; Bell-Sakyi et al. 2015). Attenuated vaccines based on the use of bacteria extracted from bovine erythrocytes are very effective, but there could be possible contamination with the erythrocyte membrane antigens (Ocampo Espinoza et al. 2006). A wide antigenic variability and genetic diversity exist among strains found within the same animal or the same herd, and this has constrained the production of a recombinant anaplasmosis vaccine (Quiroz-Castañeda et al. 2016a, 2016b).

Despite the number of resources and technological advancements in advanced vaccine approaches, molecular biology, proteomics, and biotechnology, there is still no effective vaccine against anaplasmosis because of the genetic diversity among strains of the same species (Quiroz-Castañeda et al. 2016a, 2016b). Several scientific studies have been made towards the use of advanced vaccine technology based on proteomics, metabolomics and transcriptomics to develop a suitable anaplasmosis vaccine (Quiroz-Castañeda et al. 2016a, 2016b). Epitope-based vaccine demonstrated by Santos et al. (Santos et al. 2013) using a functional motif of MSP1 and STSSxL induced a balanced humoral and cellular immune response and increased expression of proinflammatory cytokines. Hence, this vaccine could be a viable alternative to induce protective immunity against bovine anaplasmosis. Control of bovine anaplasmosis using vaccination is difficult because of the genetic variability mechanism *A. marginale* uses to evade the host immune system (Dark et al. 2012). The use of subdominant outer membrane protein (OMP) antigens as the best vaccine candidates was evaluated (Ducken et al. 2015) AM854 and AM936 induced high IgG and IgG2 responses, and were proposed to be adapted for use in subunit vaccines. The challenges encountered were the difficulty and high cost associated with the isolation of the protein antigens, making it impractical for development and implementation in vaccination programme. More ‘omics’ studies will have to be required in order to ascertain the most relevant antigens to be used eventually in a reliable vaccine for the control of anaplasmosis in most part of the world (Quiroz-Castañeda et al. 2016a, 2016b).

### Haemotropic *Mycoplasma *species

Haemotropic *Mycoplasma*s or haemoplasma (formerly *Haemobartonella* and *Eperythrozoon* spp.) are small, pleomorphic, obligate, gram-negative, uncultivable cell-wall-less bacteria found attached to the erythrocyte surface or free in the serum of animals (Neimark and Kocan 2006; dos Santos et al. 2012). They may occur in chains across the erythrocyte surface, or singly as rod-shaped, ring-shaped or spherical. They are found enclosed by a single limiting membrane and possess a few filamentous structures and small granules in the cytoplasm, without a nucleus. None of the haemoplasmas have been cultured outside their host because of their lack of a cell wall. This emerging bacterial pathogen infects a wide variety of animals, including livestock (Mohd Hasan et al. 2017; Byamukama et al. 2020), wildlife (Watanabe et al. 2010; Díaz-Sánchez et al. 2019) and companion animals (Maggi et al. 2013). A virulent form of *Mycoplasma suis* has been found in intracellular vacuoles or floating freely within infected erythrocytes, and the mode of entry seemed to begin with invagination of the erythrocyte membrane in an endocytosis-like process (Groebel et al. 2009). This method of entry into erythrocytes is the same as that of *Plasmodium falciparium* and *Bartonella bacilliformis* (Haldar and Mohandas 2007). *Mycoplasma suis* was detected in reticulocytes and normoblasts, and this implies possible replication within erythrocyte precursors in the blood marrow. *Haemobartonella* and *Eperythrozoon* spp. were classified as members of the order *Rickettsiales* based on their phenotypic and biologic characteristics, such as their small size, erythrocyte tropism, obligate parasitism, and transmission by ticks. Because of the absence of a cell wall, small genome, use of the codon UGA to encode tryptophan, 16 S rRNA gene sequences, resistance to penicillin and susceptibility to tetracycline led to increasing doubt on this classification. These organisms were found to be closely related to class Mollicutes, order *Mycoplasmatales*, and family *Mycoplasmaceae*. Phylogenetic analysis of the 16 S rRNA gene revealed low similarity with other *Rickettsiaceae* but a close relationship to *Mycoplasma* species. As a result, *Haemobartonella* and *Eperythrozoon spp.* were reclassified under *Mycoplasma*, with the *‘Candidatus’* prefix for newly identified, incompletely described species (Neimark et al. 2002).

*Haemobartonella* and *Eperythrozoon* form a distinct haemoplasma group within the genus *Mycoplasma*, characterised by their strong affinity for erythrocytes and low 16 S rRNA gene sequence similarity to other bacteria, including mucosal *Mycoplasmas*. Also, they were reported to be more closely related the *pneumoniae Mycoplasmas* than the mucosal *Mycoplasmas*. Therefore, the use of RNA subunit of the RNase P RNA (*rnpB)* gene for phylogenetical analysis was preferred since the gene is universally present in bacterial species and more suitable for phylogenetic discrimination of closely related species when compared to the 16 S rRNA gene (Peters et al. 2008). The *rnpB* gene has a high rate of nucleotide variation and encodes the RNA subunit of endoribonuclease. Thus, phylogenetic analysis based on *rnp*B gene confirmed the close relationship between *Haemobartonella* and *Eperythrozoon* spp. with *Mycoplasma* species and all haemoplasma species were located within the same clade (Peters et al. 2008).

Haemoplasmosis is usually considered to be a subclinical disease and it is associated with significant haematological disorder (haemolytic anaemia) in cattle, small ruminants and camelids (Baggenstos et al. 2012; Tagawa et al. 2013). It drastically affects immunologically naïve, immunosuppressed, or debilitated animals. In cattle, formerly only one species, *Mycoplasma wenyonii* was regarded as the causative agent of haemoplasmosis, however, based on the 16 S rRNA genotype, a novel microorganism, ‘*Candidatus’ Mycoplasma haemobos* was also discovered (Hofmann-Lehmann et al. 2004; Tagawa et al. 2008). These two bovine haemoplasmas are the major causative agents of bovine haemoplasmosis, with a worldwide occurrence (Hoelzle et al. 2011; Song et al. 2012; Tagawa et al. 2013). In camels and alpacas, the disease is caused by *Mycoplasma haemolamae* (Tornquist et al. 2011). Clinical signs associated with the bovine haemoplasmosis include mild to severe haemolytic anaemia, transient fever, prefemoral lymphadenopathy, udder, scrotal and hindlimb oedema, decrease in milk yield, tachycardia, weight loss, diarrhoea, rough hair coat, vaginal bleeding, swollen teats, low birth weight of infected calves and reproductive problems due to scrotal degeneration (McAuliffe et al. 2006; Tagawa et al. 2008; Baggenstos et al. 2012). In chronic infection, signs of anaemia or chronic ill-thrift may be present without signs of active haemolysis such as icterus (Tagawa et al. 2013). Co-infection with *Anaplasma marginale* was demonstrated to enhance anaemia and its fatal in affected cattle (Hornok et al. 2012). According to a recent study ‘*Candidatus’ Mycoplasma* haemobos appears to be more pathogenic than the *Mycoplasma wenyonii* (Tagawa et al. 2010) and cattle that recover from primary infections caused by both pathogens may remain chronic carriers (Nishizawa et al. 2010; Tagawa et al. 2010). *Candidatus Mycoplasma haemozalophi*, a novel haemotrophic *Mycoplasma* has been detected in California sea lions (Volokhov et al. 2011). The routes of infection of bovine haemoplasma are through the bite of ixodid ticks such as *Rhipichephalus microplus* and *Haemaphysalis bispinosa* in China (Shi et al. 2019) and Malaysia (Mohd Hasan et al. 2017), and *Ixodes ricinus* ticks in South-East Europe (Stevanović et al. 2020). Mechanical transmission is through bites of blood-sucking arthropods such as Rhipicephalus the horn fly (*Haematobia irritans*), the stable fly (*Stomoxys calcitrans*) and two species of horse fly, *Tabanus bovinus* and *T. bromius*) (Hornok et al. 2011). Vertical transmission through the placenta has also being reported (Fujihara et al. 2011; Hornok et al. 2011). In China, lice, flies and mosquitoes have tested positive for *Mycoplasma wenyonii* (Song et al. 2012). Risk factors associated with the disease include immune status, door housing, and age as cattle between 1–3 years of age are more susceptible *to M. wenyonii* infection than older cattle (Congli et al. 2011; Tagawa et al. 2012).

### Pathogenesis of haemotropic *Mycoplasma species* infection

Haemotropic *Mycoplasma* have yet to be cultured in vitro, and this has constrained the study of their life cycle and pathophysiological mechanism of the disease. Briefly, following the bite of the suitable arthropod vectors (ixodid ticks) or mechanical transmission by flies, mosquitoes and lice, or vertical transmission via blood contaminated needles or instruments, bacteriaemia develops within days of inoculation. In immunosuppressed animals, clinical signs of the disease develop. However, the attachment of haemoplasma onto erythrocyte surfaces leads to indentations and small depression on their surfaces. These alterations, thus leads to immune-mediated destruction of red blood cells (extravascular haemolysis) or the direct damage results in increased fragility of the erythrocytes and subsequent haemolysis, resulting in anaemia (Willi et al. 2005). Intracellular invasion of erythrocytes by *M. suis* results in cell damage and subsequent destruction of the red blood cell (Groebel et al. 2009). Immune-mediated lysis of red cells is orchestrated by increased autoreactive antibodies reported during the acute phase of haemolytic anaemia in affected animals (Tasker 2010; Gladden et al. 2016).

Felder et al. (2011) proposed a new mechanism for development of haemolytic anaemia in haemoplasmosis and used *M. suis* as a representative for haemotropic *Mycoplasma*s. Felder and co-workers observed that *M. suis* induced or triggered programmed erythrocyte cell death (eryptosis), which is characterised by cell shrinkage, micro-vesiculation and exposed phosphatidylserine on the outer red cell membrane. It was speculated that haemoplasma trigger eryptosis by consuming nutrients meant for erythrocytes thereby reducing their life span. Soluble *Mycoplasma* substances and stress signals was detected in sera of experimental pigs. Therefore, lack of energy and oxidative stress may trigger eryptosis and concluded that eryptosis is one of the anaemia-inducing factors during *M. suis* infections (Felder et al. 2011). Acute phase of haemoplasmosis is characterised by bacteriaemia, and unspecified clinical signs such as pale mucous membrane, fever, lethargy, anorexia, weight loss, depression, and dehydration. Anaemia that developed during the course of the disease may lead to hypoxemia and death may occur. After primary infection, affected animals may become healthy chronic carriers and source of infection to naïve cattle.

###  Immune response to haemotropic *Mycoplasma* species infection

The rapid onset of an effective immune response mounted against haemotropic *Mycoplasma*s curtails proliferation and stops progression of the disease in immunocompetent individuals. Furthermore, latent infection is usually common in endemic areas and the bacteria can co-exist with the host without causing the clinical signs of the disease (Baggenstos et al. 2012). However, autoreactive antibodies are targeted towards the actin and glycoprotein on the infected erythrocytes (Felder et al. 2010). There are no experimental studies in available literature to fully understand the immune response to *Mycoplasma wenyonii* and *C. M. haemobos* infection has been documented.

###  Evasion of immune system by haemotropic *Mycoplasmas*: Adaptive and survival properties

The infections caused by haemotropic *Mycoplasma*s are usually chronic, and this means that the epicellular bacteria have developed means to escape the host immunity. The inability of haemotropic *Mycoplasma*s to produce compounds required for their survival makes them heavily dependent on the host cell (Novacco 2012). The receptor by which haemoplasma adhere onto erythrocytes surfaces is yet to be defined. In humans, an overexpression of complement receptor type I (CR1, CD35), a multiple-nodular protein that plays a significant role on erythrocyte immunity has been observed in *Mycoplasma suis* infection in humans (Congbin et al. 2010). This receptor has been used by other intracellular pathogens such as *Plasmodium falciparium* to invade erythrocytes (Awandare et al. 2011), and it is suggested that CR1 may play a role in the pathogenesis of haemoplasmosis. Tissue sequestration as a means of immune system evasion by *M. haemofelis* and *C. M. turicensis* in cats has been reported but there is lack of evidence of this method of host evasion in cattle (Novacco 2012). The bacteria remain quiescent in tissues for a long-time post exposure, but tissue load appear to decrease over time (Novacco et al. 2011). This mechanism protects the bacteria from the host’s immune response and may explain the chronic nature of haemoplasmosis (Groebel et al. 2009; Novacco 2012). This is same for *M. suis* transformed into nanoparticles as a mechanism to survive for months or years within host tissue cells (Schreiner et al. 2012). Other Mycoplamas has been reported to evade the host immune system with the aforementioned mechanism (Demina et al. 2010). Reactivation of infection following tissue sequestration occur in cats with concurrent immunosuppressive conditions such as neoplasia.

*Mycoplasma wenyonii* might use recombination or phase variation as a mechanism of antigenic variation to evade the bovine immune system. This was attributed to its protein-coding sequences which about 52.6% of it were represented by hypothetical proteins and possessed a large repertoire of paralog genes (dos Santos et al. 2012). Also, molecular mimicry by surface proteins could be another means of immune system evasion.

### Haematology and serum biochemistry

A low mean corpuscular volume and mean corpuscular haemoglobin values with a high leukocyte count has been observed in *C. M. haemobos* infected cattle. Co-infections with *M. wenyonii* and *C. M. haemobos* only resulted in high leukocyte count (Niethammer et al. 2018). A markedly regenerative, severe macrocytic anaemia with spherocytosis and basophilic stippling has been demonstrated in *Mycoplasma wenyonii* infected Holstein dairy cow (Gladden et al. 2016). Regenerative anaemia with marked anisocytosis, moderate polychromasia, lymphopaenia, monocytosis and a marked increase in fibrinogen concentration were recorded for an infected *M. wenyonii* infected Angus Cow (Genova et al. 2011). Elevation in serum activities of ALP, AST, GGT and glutamate hydrogenase, and hyperbilirubinaemia (unconjugated) were the serum biochemistry associated with haemoplasmosis in infected Holstein dairy cows. Hyperbilirubinaemia without haemoglobinaemia or haemoglobinuria is an important feature of the disease. Intraerythrocytic haemosiderin accumulation in lymph node and spleen macrophages, as well as Kupffer cells, along with extramedullary haematopoiesis in lymph nodes, was observed. The increase in erythroid precursors and megakaryocytes indicates a regenerative response to anaemia. A mild decrease in magnesium concentration, elevated serum activity of lactate dehydrogenase (LDH), decreased creatine phosphokinase (CPK) activity, a profound hypoglycaemia, acidosis and increased anion gap were among the clinical chemistry abnormalities associated with bovine haemoplasmosis. Tagawa et al. (2010) reported significant decreases in mean RBC count, Hb and PCV levels in both *M. wenyonii* and *C. M. haemobos* infected Holstein-Friesian cattle herd, with that of *C. M. haemobos* infection much lower than those of *M. wenyonii*. Macrocytic anaemia and leukocytosis were observed in the affected cattle herd. Concurrent infection with *Mycoplasma wenyonii* and *Haemobartonella bovis* in indigenous Iraqi cattle was associated with reduced haemoglobin concentration, haematocrit levels, and counts of red blood cells and platelets. This condition correlated with a marked increase in white blood cell numbers, as well as neutropenia, lymphopaenia and monocytosis. (Al-badrani and Al-abadi 2016). In chronic bovine haemoplasmosis, a normal haematological parameter, except for slight anaemia, has been reported in infected cattle (Tagawa et al. 2013).

###  Diagnosis of Haemotropic* Mycoplasma* species infection

Diagnosis of haemoplasmosis is based on the traditional visual assessment of Giemsa-stained, Wright-stained or acridine-orange stained blood smears by light microscopy or fluorescence microscopy, respectively (Niethammer et al. 2018). Haemotropic *Mycoplasma*s appear as small (0.5–3 μm), blue to purple-stained coccoid, rod-shaped organisms attached onto erythrocyte surfaces. Under fluorescence microscopy, they appear as contrast-stained, dot-like deposits on erythrocyte surfaces (Niethammer et al. 2018). The diagnostic specificity of this method is affected by confusing the organisms with artefacts, Howell-Jolly bodies and stain precipitates. Also, there is an inability to differentiate between haemoplasma species. Another constraint is that if the blood smears are not made as soon as possible post-sample collection, the bacteria may detach easily and appear as stain precipitates or debris (Tornquist et al. 2010). Therefore, the detachment of the bacteria from erythrocyte surfaces, the cyclic nature of the bacteriaemia, as well as low bacteraemia often present, make microscopy insensitive (Tornquist et al. 2009). Complement fixation and indirect fluorescent antibody tests are positive for only a short period of time or within a few days of the onset of the clinical disease. A paired serology test is preferably used to distinguish between recent infection and previous exposure. Molecular analysis based on 16 S rRNA polymerase chain reaction, *rnp*B PCR or real-time PCR is better, highly sensitive and specific in the diagnosis of haemoplasmosis in comparison with the traditional microscope methods (McAuliffe et al. 2006; Peters et al. 2008; Meli et al. 2010). Thus, PCR is the gold standard method for the diagnosis of haemoplasma (reviewed in Messick 2004; Tornquist et al. 2009; Shi et al. 2019), infection and species differentiation, and has facilitated the study of the animal’s response to treatment (Messick 2004; Tornquist et al. 2009; Shi et al. 2019). The detection of a positive direct antiglobulin test confirms autoimmune-mediated haemolytic anaemia. Historically, diagnosis has been made based on the detection of organisms on routine Wright-stained blood smears, on which they appear as small basophilic, round, rod, or ring-shaped structures present on erythrocytes individually or in chains or sometimes seen free in the background. However, bacteriemia in chronic infections can be cyclic as organisms can disappear from circulation in as little as two hours. In addition, haemoplasma dissociates from erythrocytes and dies after a variable amount of time in EDTA, hampering the detection of organisms in aged samples. The recent development of sensitive PCR assays capable of discriminating between various haemoplasma has greatly enhanced the diagnosis of this pathogen and has led to the identification of several new *Mycoplasma* spp.

### Prevention and control strategies

Prophylactic antibiotic treatment using oxytetracycline, doxycycline and quinolones is effective in reducing haemoplasma loads (Dowers et al. 2009), although complete elimination has never been achieved (Genova et al. 2011). Glucocorticoids may be useful to decrease erythrophagocytosis in cases of severe haemolysis. The treatment only resolves the haematological abnormalities and improves the clinical signs associated with the disease. Vaccination is a good preventive measure, and research on potential ligands between erythrocytes and haemoplasma could mark an important step in understanding the pathogenic mechanism of haemoplasma and may be useful for vaccine development in the future (Novacco 2012). Control of arthropod vectors by the use of acaricides is recommended, as is minimising stress in herd and flock situations.

### *Trypanosomaspecies,*with emphasis on *Trypanosoma evansi*

Trypanosoma is a genus within the family *Trypanosomatidae*, order *Kinetoplastida*, and subgenus *Trypanozoon*.(Duarte et al. 2018). These protozoan parasites have been associated with significant socio-economic impact and limit animal productivity in endemic parts of the world (Desquesnes et al. 2013). They are blood and sometimes tissue parasites transmitted by haematophagous biting flies, in which most of them undergo a biological cycle. Trypanosomes are unusual protozoan parasites in that, in the host, they are surrounded by a thick immunogenic surface coat referred to as the variant surface glycoprotein (Namangala 2012).They are divided into two main sections, dependent on the part of the digestive tract where the protozoa develop. They include the Stercoraria, which develop in the posterior part of the insect digestive tract, e.g. *Trypanosoma cruzi*, the causative agent of Chagas disease (reviewed in Coura and Borges-Pereira 2010), and Salivaria, which develops in the anterior part of the insect digestive tract, e.g. the African pathogenic trypanosomes (*Trypanosoma brucei*) (Simwango et al. 2017).

Trypanosomosis is a group of diseases caused by parasitic trypanosomes and responsible for serious production losses in the livestock industry in Sub-Saharan Africa (Duarte et al. 2018). Trypanosomes are extracellular, microscopic and haemoflagellate protozoan parasites belonging to the genus *Trypanosoma* that survive in the bloodstream. This haemoprotozoan is virulent, inoculable, but not contagious (except *Trypanosoma equiperdium*). These parasites are commonly found in the tropics and can affect a wide variety of domestic and wild animals, including humans. It causes high morbidity and high mortality in infected herds, thereby making the disease very difficult to manage in endemic areas (Mijares et al. 2010). Parasitic trypanosomes of veterinary importance include *Trypanosoma vivax*, *T. congolense*, *T. brucei*, *T. equiperdum* and *T. evansi*. These trypanosomes are cyclically transmitted by tsetse flies except for *T. evansi*, which is mechanically transmitted by biting flies such as *Tabanus* species. Cattle develop patent infections depending on the strain, species and number of infecting trypanosomes introduced. A disease condition known as Nagana (African animal trypanosomosis) is caused by *T. congolense* and *T. brucei* in cattle. *T. brucei* is also a causative agent of the human sleeping sickness in Africa (Lai et al. 2008). Nonspecific clinical symptoms associated with trypanosomoses include anaemia, fever, weakness, nervous signs, infertility, cachexia, lymphadenopathy, abortion and death if left untreated. Of all the trypanosomes, *T. brucei* is highly virulent and characterised by the development of subcutaneous oedema of the ventral abdominal wall, thorax and limbs, keratoconjunctivitis, ataxia and paralysis. *Trypanosoma vivax* or *T. congolense* usually produces the chronic form and may cure spontaneously in local breeds (Taylor and L-Authie 2004). Animal and human trypanosomoses have threatened about 48 million cattle and 60 million people in Africa (Rodgers 2009; Desquesnes et al. 2013). Cattle production losses are estimated to surpass USD 12,000 million annually (Namangala 2012).

*‘*Surra’ or ‘mal de cadeiras’ caused by *Trypanosoma evansi* (a euryxenous parasite), is an economically significant disease that affects a wide range of domestic and wild animals including cattle, buffaloes, sheep, goats, pigs, horses, donkeys, camels, dogs, deer, gazelles and elephants in some parts of Europe, South America, Asia, Africa, Central America. It’s characterized by high morbidity and mortality (Desquesnes et al. 2013). *Trypanosoma evansi* is a salivarian trypanosome transmitted mechanically from one susceptible host to another by blood-sucking flies belonging to the genera *Stomoxys* and *Tabanus* (Ereqat et al. 2020). Vampire bats (*Desmodus rotundus*) may serve as host, reservoir or biological vectors of *T. evansi* in Latin America (Carnes et al. 2015). Oral transmission to carnivores when feeding on a fresh infected carcass has been reported (Sinha et al. 1971). Transplacental transmission of *T. evansi* from a challenged donkey mare to a neonatal foal has also been reported (Kumar et al. 2015). *Trypanosoma evansi* is known to cause persistent infections of the blood and induce immune suppression. The clinicopathological signs and lesions of the disease include anaemia, cachexia, decrease in milk and meat production, subcutaneous oedema, lethargy, progressive weight loss, nasal and ocular bleeding, stiffness of the limbs, lymphadenopathy, fever, subcutaneous oedema, nervous signs due to neuronal degeneration and meningoencephalitis, enlarged spleen with hypertrophy of lymphoid follicles, abortion and death (Jittapalapong et al. 2009; Desquesnes et al. 2013; Gutiérrez et al. 2014; Acosta et al. 2016). *Trypansoma evansi* exhibits itself as a bloodstream trypomastigote, however, it has also been found in cerebrospinal fluid and is known to invade tissues such as the kidney, brain, bone marrow, liver and spleen (Tejero et al. 2010) and has a special preference for connective tissues and fibroblasts (Acosta et al. 2016). It originated from Camels in Africa (Diall et al. 1993) and has spread far beyond its primary territory (tsetse belt) in Africa to Southeast Asia (Adrian et al. 2010; ElShafie et al. 2013), Central and South America (Gutierrez et al. 2005) and recently in Europe (Desquesnes et al. 2008; Tamarit et al. 2010; Bono Battistoni et al. 2016; Greif et al. 2018), because of its adaptation to mechanical transmission.

Previously, *T. evansi* was considered non-infective to humans, but human infections have been confirmed in Vietnam (Van Vinh Chau et al. 2016) and India (Joshi et al. 2005; Powar et al. 2006; Truc et al. 2007), indicating that *T. evansi* may be an emerging human pathogen. It’s physiologically related to *T. brucei* but possesses minor differences on the subcellular level (Carnes et al. 2015). It is often regarded as a petite mutant of *T. brucei* (Lai et al. 2008). *Trypanosoma evansi* strains have either kinetoplastic DNA (kDNA), without maxicircles or minicircles (dyskinetoplastic), or completely lacking kinetoplastic DNA (akinetoplastic) (Lai et al. 2008; Birhanu et al. 2016). Those possessing kDNA minicircle type are classified into Types A and B (Masiga and Gibson 1990). A fundamental genetic distinction between the two types is the presence of the gene encoding the RoTat1.2 VSG in Type A and its absence in Type B (Ngaira et al. 2005; Birhanu et al. 2016). The epidemiological distribution of Type B *T. evansi* is notably limited; current data indicate it is both less frequent and endemic to Kenya (Ngaira et al. 2005), Ethiopia (Hagos et al. 2009; Birhanu et al. 2016) and Sudan (Salim et al. 2011), while the Type A has a widespread distribution. The absence of a functional mitochondrion could be the reason for their permanent presence in the circulating blood and relies on the glycolytic pathway as a source of ATP, as glycolysis is the sole source of ATP in all bloodstream forms of African Trypanosomes (Bringaud et al. 2006). It is worthy of note that *T. evansi* has only a trypomastigote bloodstream form and requires constant modification of the variant surface glycoprotein (VSG) coat to evade the host immune system (Duarte et al. 2018). Also, due to a loss of genetic material, *T. evansi* can no longer undergo its cycle in tsetse flies; this is the reason for its mechanical transmission in a susceptible host (Desquesnes et al. 2013). Different cattle breeds respond to trypanosomosis in different ways. In contrast to zebu breeds, West African taurine cattle like the N’Dama, exhibit natural resistance to trypanosomosis and demonstrate rapid recovery following infection. This trait, referred to as trypanotolerance, distinguishes them from Boran cattle (*Bos indicus*), a Zebu-type breed, which are considered trypanosusceptible. O’Gorman et al. (2009) demonstrated in a cytokine mRNA profiling study that following infection with *T. congolense* in cattle, the trypanotolerant N’Dama cattle breed displayed a more rapid and distinct transcriptional response to trypanosome infection with a ten-fold higher number of genes differentially expressed at 14 days post-infection compared to trypanosusceptible Boran cattle. The products of the expressed genes encoding pro-inflammatory cytokines (IFN-gamma, tissue necrosis factor, and IL-12) may have contributed to a more robust response to parasites and stress, and to a heightened B cell response in N’Dama cattle. At peak parasitaemia, the trypanosusceptible cattle had a sustained T helper type 2 cytokine (IL-6 and IL-10) response. Thus, a trypanotolerant cattle possesses traits that enable it to respond very early in infection, control parasitaemia effectively and limit anaemia development and progression (Naessens 2006). Taiwo and Ogunsanmi (2000) linked the high serum erythrocyte surface sialic acid concentration in N’dama cattle to their trypanotolerance compared with trypanosusceptible Zebu cattle.

###  Life cycle of *Trypanosoma evansi*

The *T. evansi* is transmitted mechanically to animals through the bite of *Tabanus* and *Stomoxys* flies. The parasite does not have the procyclic and metacyclic tse-tse fly stages except the bloodstream trypomastigote stage because of their lack of kinetoplastic DNA (kDNA), and therefore do not undergo cyclical transmission in the tsetse fly (Carnes et al. 2015). *Trypanosoma evansi* strains possess a free flagellum and a small sub-terminal kinetoplast and multiply in the mammalian host by longitudinal binary fission. The chances of successful transmission of *T. evansi* from one host to another depend on how short the interval between two feedings of the mechanical vector. This is because the parasite cannot survive more than 15 min in the proboscis of the Tabanus fly.

**Fig. 2:**
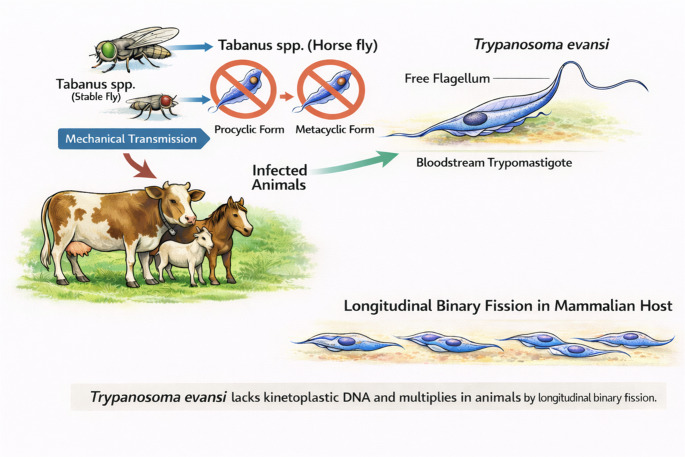
The life cycle of *Trypanosoma evansi*

It is believed that *T. evansi* is derived from *T. brucei* by deletion of a genetic material, known as maxicircle kinetoplastic DNA, which is required for cyclical development in tsetse flies, and thus, adapted to mechanical transmission (Carnes et al. 2015). Therefore, no cyclical development occurs. Transmission of *T. evansi* by vampire bats is also not cyclical because the trypanosomes multiply in them only as bloodstream forms, and developmental tsetse fly stages are not present in these mechanical vectors. *Trypanosoma evansi* is primarily characterized as morphologically monomorphic, meaning that it predominantly exists in the trypomastigote stage, which is composed of long, slender forms. However, it is important to note that intermediate and short stumpy forms may also be observed in certain cases. As a result, although the majority of *T. evansi* strains exhibit monomorphism, some strains are described as pleomorphic due to the rare occurrence of these alternative morphological forms (Misra et al. 2016) (Fig. 2).

###  The pathogenesis of *Trypanosoma evansi* infection

The first sign of *T. evansi* infection is skin swelling, known as a chancre, where the multiplying parasites are found. The skin swelling is followed by a high fever, the body’s response to the fulminating parasitaemia. Activities of the trypanosomes result in progressive anaemia, which is caused by extravascular haemolysis. Haemolysis may be triggered by an immune-mediated reaction against the red blood cells. Recurring episodes of fever and parasitaemia occur as the disease progresses alongside the loss of body condition and lassitude associated with trypanosomosis. In the chronic stage of the disease, there may be oedema of the lower parts of the body. Urticarial plaques and petechial haemorrhages of the serous membrane are often observed. Skin rashes on the ears and lateral side of the body may be seen, and abortion may occur. The escape of *T. evansi* into the central nervous system has been documented. Nervous signs associated with the diseases make the disease vary from chronic to acute and fatal, with clinicopathological signs such as recurrent fever, lymphadenopathy, emaciation, weakness and death (reviewed in Habila et al. 2012). Following the establishment of infection, the trypanosomes become a target for antibody-mediated destruction and are cleared from the blood. However, the remaining parasites survive and establish the infections due to the new variant surface glycoprotein (VSG). The repeated antigenic change of VSG in trypanosomes allows them to evade thymus-dependent response, resulting in successive surges of parasitaemia, a situation similar to being successively infected but by related but not identical pathogens. This phenomenon makes it difficult to develop a vaccine against the disease. During trypanosomosis, a membrane-associated phospholipase C enzyme cleaves the membrane-bound variant surface glycoprotein (mfVSG) from the parasite surface. This action releases a soluble VSG (sVSG) into the extracellular environment, which retains the glycosylinositolphosphate (GIP) moiety but leaves the dimyristoylglycerol (DMG) anchor within the parasite membrane. (Namangala 2012). Hyperactivation of the complement system via the classical pathway has been reported to play a significant role in immunopathology that occurs in trypanosomosis, including glomerulitis and thrombosis (Namangala 2012). The centre of immunopathology in trypanosomosis is the profound dysregulation of the macrophage system, T-cell independent production of antibodies to the VSG of trypanosomes and the anti-VSG antibody-mediated phagocytosis of trypanosomes by macrophages (reviewed in Awukew et al. 2017).

### Responses of the innate and adaptive immune systems to trypanosome infection

In the mammalian host, the trypanosome parasite must face the host immune system which is constantly trying to control the infection (Silvester et al. 2017). The immune response to infecting trypanosomes is complex and involves both the humoral and cellular parts of the immune system. These protozoan parasites live in the blood and lack the intracellular stages, therefore making the parasite a target for antibody-mediated destruction. Most trypanosomes are cleared from the peripheral circulation by macrophages of the liver (reviewed in Zambrano-Villa et al. 2002). Monocytes, macrophages, certain subsets of dendritic cells, and granulocytes constitute the primary components of the innate immune system that respond immediately to trypanosome infection. These cells serve as the first line of defence, acting rapidly to recognise and attempt to eliminate the invading trypanosomes and their associated products. Through various mechanisms, including phagocytosis and the release of inflammatory mediators, these innate immune cells help to control parasite load during the early stages of infection and initiate the inflammatory response that shapes subsequent adaptive immunity (Mansfield and Paulnock 2005). These immune cells, particularly those of the innate and adaptive immune systems, play a pivotal role in determining whether an animal is tolerant or susceptible to trypanosomosis. The interplay between these cells and the host’s immune response is crucial for survival during trypanosome infection (Gómez-Rodríguez et al. 2009). Many immunomodulatory cytokines, such as IL-12, IL-6, IL-10, IL-4, TNF-α, and IFN-γ, are produced during the course of the infection following macrophage activation by shed VSG and membrane form VSG of the protozoa. Macrophages play a pivotal role in the host’s defense against trypanosome infection, acting both as initiators of adaptive immune responses and as key effectors in T cell-mediated immunity. Their activation is a signature event during the course of trypanosomosis in cattle. Upon activation, macrophages secrete a range of proinflammatory cytokines. These cytokines are crucial, as they stimulate a robust T cell response directed against trypanosome antigens, which persists throughout the infection period. This process ensures that the immune system remains engaged in combating the parasite’s presence. The activation of macrophages during trypanosomosis is triggered by the interaction between the glycosylphosphatidylinositol (GPI) membrane anchors of the parasite’s variant surface glycoprotein (VSG) molecules and the pathogen recognition receptors on the macrophage surface. These receptors are specifically designed to detect pathogen-associated molecular patterns, including CpG DNA, thereby facilitating the recognition and response to trypanosome infection.

The cytokine environment during trypanosomosis influences the development of two main macrophage subsets namely: classically activated macrophages (M1) that develop in early phase of trypanosomosis in a type-1 cytokine (TNF-a, IL-12, IFN-y) environment, and alternatively activated macrophages (M2) that develop during the late phase of trypanosomosis in a type-II cytokine (IL-4, IL-13, IL-10, TGF-b) environment. The cytokine milieu present during the course of trypanosomosis plays a pivotal role in determining the differentiation of macrophage subsets within the host. As the infection progresses, two principal types of macrophages emerge, each associated with distinct phases and cytokine profiles. During the early phase of trypanosomosis, the host immune system generates a type-1 cytokine environment characterized by the presence of pro-inflammatory cytokines such as tumor necrosis factor-alpha (TNF-α), interleukin-12 (IL-12), and interferon-gamma (IFN-γ). This specific cytokine profile promotes the development of classically activated macrophages, known as M1 macrophages. M1 macrophages are associated with robust inflammatory responses and play a critical role in the initial immune defense against the invading trypanosomes. As the disease transitions into its late phase, the cytokine environment shifts towards a type-II profile. This environment is dominated by anti-inflammatory and immunoregulatory cytokines, including interleukin-4 (IL-4), interleukin-13 (IL-13), interleukin-10 (IL-10), and transforming growth factor-beta (TGF-β). The presence of these cytokines induces the differentiation of alternatively activated macrophages, referred to as M2 macrophages. M2 macrophages are involved in tissue repair, resolution of inflammation, and regulation of immune responses during the chronic or recovery stages of trypanosomosis (Namangala 2012). Furthermore, it has been suggested that M1 macrophages contribute to trypanotolerance mainly during the early stages since they possess pro-inflammatory properties and secrete nitric acid, reactive oxygen species and TNF-α, which help in the control of parasite growth. M2 has been associated with trypanosusceptibility because of its anti-inflammatory properties and induction of increased arginase activity (Baetselier et al. 2001), and trypanotolerance by aiding tissue repair and promoting peripheral tolerance, while suppressing harmful M1 and type 1 cytokine responses (Stijlemans et al. 2010).

A factor known as trypanosome-suppressive immunomodulating factor (TSIF) has been implicated in M1 activation and has recently been linked to trypanotolerance. (Gómez-Rodríguez et al. 2009). This factor triggers pro-inflammatory immune responses in the early phase of infection. The timing of macrophage differentiation and cytokine profiles is crucial for controlling trypanosome infection. If M2 macrophages and type-II cytokines develop too early, they can suppress the pro-inflammatory M1 response needed to clear parasites, weakening initial immunity and allowing trypanosomes to persist (Namangala et al. 2001). Activated natural killer (NK) cells facilitate trypanotolerance by exerting direct cytotoxic effects on trypanosomes and by mediating the secretion of cytokines and chemokines. These cells release interferon-gamma (IFN-γ), which induces the differentiation of naïve macrophages into the M1 phenotype, thereby promoting a type-1 immune response associated with trypanotolerance during the early phase of trypanosomosis. Activated natural killer (NK) cells are vital for trypanotolerance during trypanosome infection. They control parasite growth through direct cytotoxicity and by releasing cytokines and chemokines, notably interferon-gamma (IFN-γ). IFN-γ promotes the differentiation of naïve macrophages into pro-inflammatory M1 macrophages, fostering a type-1 immune response essential for managing infection. Overall, NK cells help establish an early immune environment that supports effective defense against trypanosomes (Namangala 2012). Trypanotolerance and immunomodulation have also been associated with non-VSG invariant antigens (cryptic VSG epitope, heat-shock protein and cysteine protease), which have no direct correlation to the control of parasitaemia but have been shown these antigens induce a predominant IgG response, and therefore suggest their immunomodulatory effect.

There are serum trypanolytic factors in animal and human blood that confer innate immunity in the early stages of the trypanosome infection. The trypanolytic activity in human serum is principally mediated by two proteins: apolipoprotein L-1 (APOL-1) and haptoglobin-related protein (HPR) (Wheeler 2010). Lysis of the trypanosome is induced by these factors through the creation of ionic pores in the lysosomal membrane, an event that triggers osmotic disruption and the destruction of the parasite. The formation of these pores leads to fatal osmotic and ionic imbalances within the lysosome, ultimately triggering the death of the parasite through uncontrolled shifts in ion concentrations and osmotic pressure. A study proved that humans with *T. evansi* infection were found to lack the APOL-1 and hence their susceptibility to the infection (Vanhollebeke et al. 2006). Cape buffaloes have a natural resistance to trypanosome infections due to trypanocidal factors in their serum, mainly hydrogen peroxide produced during purine breakdown by xanthine oxidase. This process helps neutralise trypanosomes and limits infection, making Cape buffaloes more tolerant than other susceptible animal species (trypanocidal serum protein). Therefore, a study further confirmed a 10-fold increase in serum xanthine oxidase when compared to domestic cattle that are susceptible to trypanosomosis (Wang et al. 2000). The complement system has been documented to play a role in trypanotolerance (Namangala 2012). Complement protein 3 (C3) mediated mice’s natural resistance to *T. lewesi*, as depletion of this complement protein rendered the mice susceptible to *T. lewesi* following challenge (Namangala 2012). It has been shown that murine C3 acts as an important opsonin, promoting the effective removal of *T. congolense* through phagocytosis. (Pan et al. 2006). During the initial stages of trypanosomosis, the secreted components C567, C3a, and C5a complex function as chemotactic factors, recruiting phagocytic cells to the site of infection. Activation of the complement system via the alternative pathway has been reported to hinder the lysis of trypanosomes, indicating that these parasites are capable of modulating host immune responses to enhance their survival. Specifically, the variant surface glycoprotein (VSG) present on the surface of trypanosomes is integral to this process. During infections caused by Trypanosoma brucei and Trypanosoma congolense, The VSG surface coat functions as a defensive barrier by preventing the initiation of the alternative complement cascade. This inhibition helps protect the trypanosomes from complement-mediated destruction, allowing them to persist within the host bloodstream and evade immune clearance.

Trypanosome clearance from the bloodstream was made possible by a complement system activated via the classical pathway through antibody-mediated trypanolysis and phagocytosis. The mannan-binding protein (MBP) pathway of complement activation is thought to be important during the early phase of trypanosomosis. This hypothesis is based on the ability of serum lectins and ficolins to bind to mannose residues present on the variant surface glycoprotein (VSG) of trypanosomes. The interaction between these serum proteins and the mannose residues can potentially trigger the MBL pathway, resulting in activation of the complement system. Such activation may contribute to the initial immune defence mechanisms against trypanosome infection by promoting opsonization and clearance of the parasites from the host bloodstream (Namangala 2012). The innate immune response is initiated when receptor-mediated monocyte activation prompts the secretion of pro-inflammatory cytokines, thereby inducing inflammation and directing subsequent adaptive immunity. Monocytes play a key role in early innate immunity by recognizing pathogens through receptors and releasing pro-inflammatory cytokines. These cytokines trigger inflammation, recruit additional immune cells, and help connect innate and adaptive immune responses for effective pathogen defence.(Mansfield and Paulnock 2005).

**Fig. 3:**
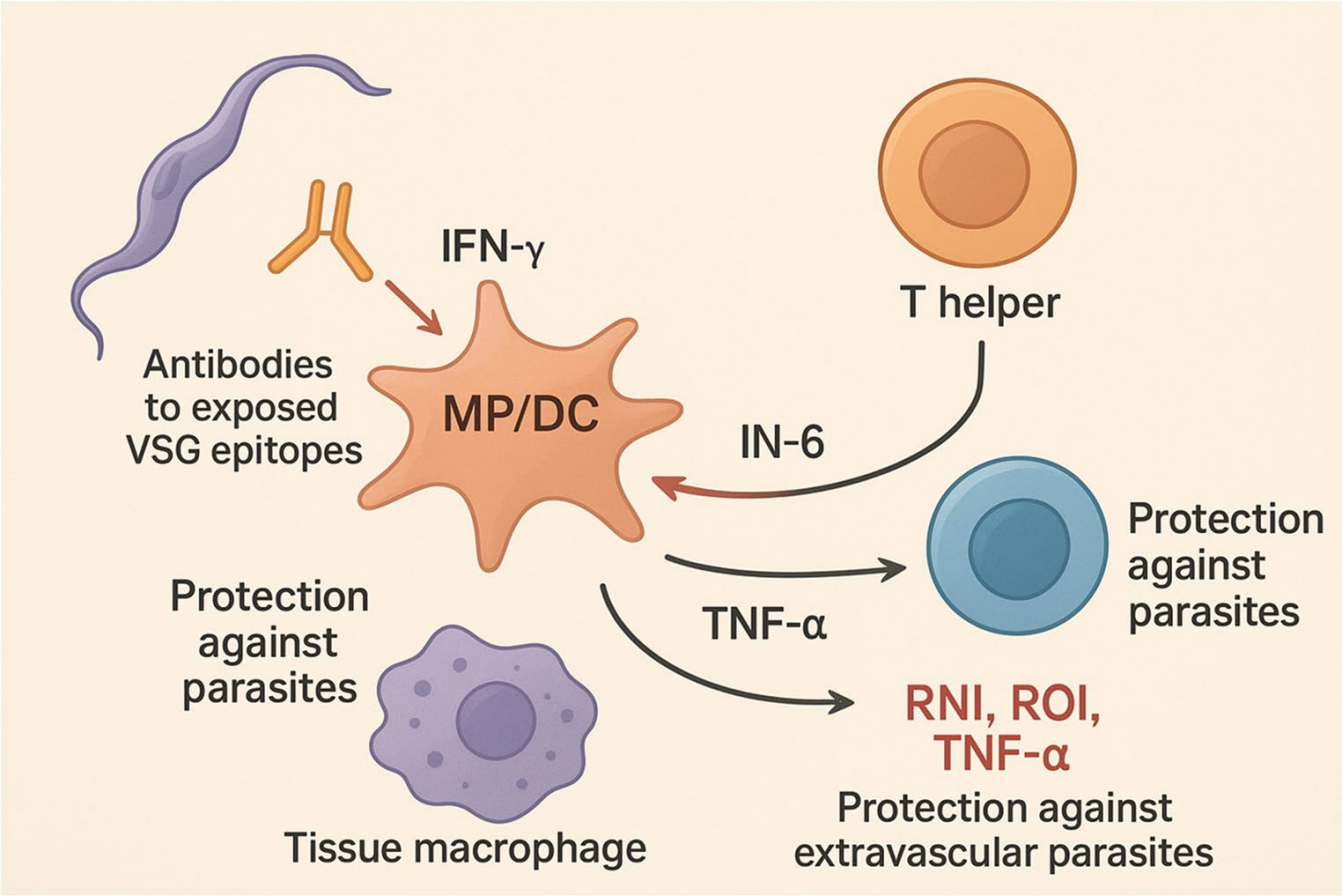
Acquired immunity to trypanosomes is largely shaped by the interaction of the host antigen-presenting cells (APCs) with trypanosome VSG and CpG DNA in the early course of the infection. The trypanotolerant cattle mount strong B and T helper 1 responses to VSG

The host immune responses to trypanosome infection include both B and T cell responses to VSG molecules that comprise the surface coat structure. The adaptive immune responses have a great impact on parasite survival in the vascular and extravascular tissue compartments of the infected animal. The extent of these responses largely depends on how trypanosomes and their molecules interact with innate immune cells (Fig. 3). Trypanosomes activate both the T-helper cell-independent and dependent B-cell responses to the VSG molecule, with the result that antibodies to exposed VSG epitopes control the growth of trypanosomes in the bloodstream and efficiently clear the protozoan parasite (Mansfield and Paulnock 2005). The cellular immune response is primarily targeted toward B cells, as T helper cells are activated by the presentation of VSG peptides via MHC II molecules on antigen-presenting cells, such as macrophages and dendritic cells. This activation induces a strong type 1 cytokine response, notably the release of IFN-γ, which in turn stimulates tissue macrophages to generate trypanocidal factors including reactive nitrogen intermediates (RNI), reactive oxygen intermediates (ROI), TNF-α, and potentially other molecules that can eliminate trypanosomes within blood and tissues.

Trypanosomes require approximately 4–5 days to replace their VSG coats fully, and it has been demonstrated that parasites undergoing coat replacement (non-switched trypanosomes) are vulnerable to clearance via early IgM antibodies for a limited time but at the early stages of coat replacement, IgM loses its ability to mediate trypanosome clearance because of the density threshold of its cognate VSGs on the parasite surface (Pinger et al. 2017). Trypanosomes that express multiple VSGs during an infection are rapidly eliminated by the host immune system, as a multi-VSG antibody response is produced, which rapidly reduces parasitaemia in mice (Aresta-Branco et al. 2019). Trypanosomes, e.g. *T. brucei*, genome encodes more than 2000 VSG genes and pseudogenes, but only expresses VSGs in a monoallelic manner, i.e. only one VSG is transcribed at a time in bloodstream types, but exposing multiple VSGs during an infection is highly detrimental to the parasite (Cross et al. 2014). Therefore, Aresta-Branco et al. (2019) suggested that drugs that disrupt the VSG monoallelic expression can be used for the treatment of trypanosomosis. Acute phase proteins such as C-reactive proteins, haptoglobin, alpha 2-macroglobulin, and IgM have been suggested to be involved in host defence against *T. evansi* in rabbits and possibly participate in the control of the protozoan parasite (Costa et al. 2013).

### Evasion of the immune system: adaptive and survival properties of trypanosome parasites

Trypanosomes are always in contact with the host immune system post-infection, and these extracellular protozoan parasites are known for their ability to avoid elimination by the immune system and thrive extracellularly within the mammalian host bloodstream by periodically switching and expressing their immunodominant variant surface glycoprotein (VSG) coat during infection, a process called antigenic variation (Pinger et al. 2017). These protozoan parasites must evade immune attack to ensure their survival and avoid excessive exploitation of host resources to ensure the host survives long enough to ensure successful transmission to the fly vector. Thus, a typical trypanosome infection is characterized by undulating waves of parasitaemia due to the periodic destruction of antigenic variants, followed by the outgrowth of antigenically distinct variants. The variant surface glycoprotein (VSG) coat covering the membrane of African trypanosomes consists of a densely packed array of 10^7^ identical molecules that determine the antigenic phenotype of the parasite. VSG molecules are 55–65-kDa glycoproteins, and these molecules are displayed as homodimers. Antigen variation is one of the several mechanisms by which these parasites thrive in the face of immune defences. Trypanosomes evade the host’s immune system by regularly replacing their surface proteins, or variant surface glycoproteins (VSGs), through controlled genetic mechanisms. Only one VSG type is displayed at a time, allowing the parasite to avoid antibodies targeting previous VSG variants and maintain ongoing infection. In other words, surface antigens are regularly shed and replaced by new ones. Other mechanisms of parasite survival include the ability to avoid complement-mediated destruction and thrive in a high level of interferon-gamma. Studies have also shown that trypanosomes that express a mosaic variant of the surface glycoprotein coat easily escape detection by the immune system and fail to activate B cells until VSG coat homogeneity is achieved directly (Dubois et al. 2005). This process is an immunological advantage to trypanosomes during the process of antigenic variation.

*Trypanosoma evansi* can invade the central nervous system, causing necrotising encephalitis, and since trypanocidal drugs do not cross the blood-brain barrier, *T. evansi* in the CNS survives anti-trypanosomal therapy. The mechanism by which *T. evansi* invades the blood-brain barrier is not clear, but it is assumed that invasion of the blood-brain barrier is disrupted either directly by the parasites or by the release of chemical mediators, including cytokines and proteases (Acosta et al. 2016).

### Evasion of innate immune response by *Trypanosoma evansi*

Parasite-derived factors, including the glycosylphosphatidylinositol (GPI) membrane anchor residues present in shed soluble VSG (sVSG), and membrane form VSG (mfVSG) molecules, and the release of CpG DNA, and host factor, Interferon-gamma, play key roles in the activation, modulation and control of many aspects of the host innate and adaptive immunity in trypanosomosis (Mansfield and Paulnock 2005). Both sVSG and mfVSG have similar macrophage-activating abilities in terms of inducing TNF-alpha, IL-6 and nitric oxide production. Macrophage activation in trypanosomosis is partially dependent on exposure of the cell membrane to sVSH and the uptake of mfVSG. Macrophage activation in trypanosomosis relies partly on exposure to soluble and membrane-bound variant surface glycoproteins (sVSG and mfVSG). These interactions trigger macrophage differentiation, supporting a pro-inflammatory response that helps control and eliminate the parasite early in infection. Trypanosomes evade host immunity by triggering a switch from pro-inflammatory M1 to anti-inflammatory M2 macrophages as infection progresses. While early M1 activation supports parasite clearance, the later dominance of M2 suppresses this response, allowing parasites to persist. This modulation of macrophage polarisation enables trypanosomes to survive and maintain chronic infection by weakening the host’s immune defences (Baetselier et al. 2001). The switch from pro-inflammatory M1 to anti-inflammatory M2 macrophages during trypanosome infection is associated with regulatory T cells and their production of IL-10. IL-10, mainly produced by Treg cells, promotes M2 gene expression, driving the immune response from a type-1 to a type-2 state. (Bosschaerts et al. 2010).

Another interesting strategy of *T. evansi* to evade the immune system is to take advantage of the host fibrinolytic system, involved in the breakdown of fibrin and extracellular matrix during tissue remodelling (Bergmann and Hammerschmidt 2007). Most pathogens require certain strategies to invade a tissue host, such as expressing surface receptors that bind plasminogen or plasmin. Acosta et al. (2016) demonstrated that *T. evansi* can bind plasminogen and plasmin on its surface, and this interaction plays an important role in the establishment of the infection in the host. *Trypanosoma evansi* makes use of a fibrinolytic enzyme, known as plasmin, a serine-protease product of plasminogen activation. The proteolytic activity of plasmin causes tissue damage and encourages pathogen invasion of tissues (Acosta et al. 2016). Therefore, *T. evansi* coated with plasminogen/plasmin can invade the CNS through degradation of extracellular matrix and removal of opsonising complement factors and immunoglobulin, degradation of fibrin that is formed around the parasite, and prevent its lysis following the classical route of complement activation (Acosta et al. 2016).

###  Evasion of innate immune response by *Trypanosoma evansi*

Successful survival of trypanosomes in the mammalian host depends on evading the host immune system by modulating the host immune response. Immunosuppression is usually caused directly by the products secreted by the trypanosomes. One of the complicated mechanisms of host immune evasion is its selective activation of a subset of T helper cells (Zambrano-Villa et al. 2002). *Trypanosoma brucei* avoids the immune system by altering the T and B-cell populations, inducing changes in the pattern of cytokines produced by CD8 + Tcells, and by production of a gp63-like protein, while *T. cruzi* causes anergy of T cells and inhibits the production of IgM antibodies in humans. The increases in IFN-γ with consequent decreases in IL-2 and IL-2R render T cells unresponsive and impairs proliferative T-cell response to avoid parasite elimination. Production of gene coding glycoprotein 63 (gp63)-like protein by the trypanosomes makes the parasite resistant to complement-mediated destruction, and thus, evades the adaptive immune system. Antigenic variation by VSG evades the previously established immune response, and alteration of the T and B cell population induces immunosuppression (Zambrano-Villa et al. 2002). Despite the effectiveness of the anti-VSG-specific antibodies, complete elimination of trypanosomes is hampered by the rapid appearance of those with different VSG to which the host immune system is yet to mount an immune response. The persistence of trypanosomes in blood circulation leads to continuous stimulation of the host immune system, as evidenced by a marked increase in size and activity of germinal centres, with a concomitant increase in proliferating lymphocytes in the medullary cords, paracortex of lymph nodes, and peripheral follicular areas of the spleen, apparent overstimulation of the humoral immune system leads to increased levels of IgM and IgG, which about 85–100% of them are absorbed by the trypanosomes. The B cells are one of the targets of immunosuppression as evidenced by the reduction in IgG1 and IgG2. Macrophages have been demonstrated to play a role in immunosuppression as they are involved in the removal of cells such as CD5 + B cell lineage and granulocytes that express Mac-1. A 40–45 kDa protein derived from *T. brucei brucei* has been demonstrated to cause a down-regulation of interferon-gamma secretion by CD8 + T cells and upregulation of IL-2R expression on both CD8 + and CD4+. A decrease in IFN-γ will hinder the stimulation of macrophage activity and suppress surface expression of MHC class I and II on various cell types.

### Haematology and serum biochemistry

Anaemia of the haemolytic type is a common feature of trypanosomosis. Haemolysis of red blood cells with decreased life span and extensive erythrophagocytosis is responsible for the low number of erythrocytes below the reference range. Anaemia is usually severe due to the level and initial wave of parasitaemia. Haemolytic factors (haemolysin and free fatty acids), immunologic mechanisms, haemodilution, coagulation disorders, depression of erythropoiesis and release of trypanosomal sialidase all play a role in the development of anaemia in trypanosomsis (reviewed in Habila et al. 2012). Sialidase cleaves the sialic acid which are present on the erythrocyte surface, and thus, exposes the galactosyl residues which are recognized by D-galactose specific lectins on macrophages leading to erythrophagocytosis by the activated and expanded mononuclear phagocytic system (Sallau et al. 2008). Trypanosomes also release phospholipases which hydrolysis the host erythrocyte membrane. All these events are associated with haemolysis that decreases the life span of erythrocytes, leading to anaemia. Oxidative stress in *T. evansi* infection occurs when cell membrane-altered or ‘damaged’ erythrocytes produce reactive oxygen species that enhance lipid peroxidation, which contributes to erythrocyte lysis and progression of anaemia in *T. evansi* infection. Haematobiochemical profile of animals infected by trypanosomosis include macrocytic hypochromic anaemia, leukopaenia, thrombocytopaenia, increased erythrocyte sedimentation rate, monocytosis, increased serum activities of ALT and AST, hypoglycaemia, elevated blood urea nitrogen, hypoalbuminaemia and hypergammaglobulinaemia, fever, anorexia, dullness, weight loss, tissue damage, immunosuppression and in some cases, death (Mijares et al. 2010; Agina and Ihedioha 2016; Hussain et al. 2018).

A profound decrease in Hb, PCV and an increase in AST activity, lipid peroxidation, increased superoxide dismutase (SOD) and catalase CAT) activities were haemato-biochemical and oxidative stress status in buffaloes naturally infected with *T. evansi* (Pandey et al. 2015). Leukocytosis, thrombocytosis, an increase in serum AST activity and serum iron, and a decrease in haemoglobin, haematocrit and serum glucose were observed in *T. evansi-infected* cattle and buffaloes found in low-lying areas of Punjab province, India (Sharma et al. 2019). Haematological findings in Nili Ravi buffaloes include decreased erythrocyte count, Hb, haematocrit, MCHC, high serum protein level with increased MCV, high total leukocyte count, monocytosis, neutrophilia, basophilia and eosinophilia. Significant increases in malondialdehyde (MDA) and serum enzymes, with a decrease in macrominerals and trace minerals (copper), were recorded in the naturally infected *T. evansi* buffaloes (Hussain et al. 2018).

###  Diagnosis of *Trypanosoma evansi* infection

Diagnostic detection of *T. evansi* employs various microscopic, serological, and molecular methods, each with different capacities to identify the two parasite types. Among these, the conventional technique of Giemsa-stained blood smear microscopy is frequently utilised to diagnose acute infections involving either type, but it is unsuitable for detecting latent or chronic cases (ElShafie et al. 2013). While microscopic examination for *T. evansi* is reliable, its utility is limited by its time-consuming nature, dependence on a light microscope, and need for skilled personnel at the point of use. These constraints were largely overcome by the development of the *T. evansi* Card Agglutination Test (CATT/T. evansi). This test has become a standard field diagnostic, particularly for *T. evansi* Type A, as it employs the RoTat 1.2 VSG antigen to detect specific host antibodies through agglutination. However, this serological test cannot differentiate between current, previous or repeated exposure (Hassan-Kadle et al. 2019). Two or more detection methods, such as mouse inoculation, haematocrit concentration technique, buffy coat test, and Giemsa-stained thin blood smear, can be combined to increase the sensitivity in the detection of Trypanosomes (ElShafie et al. 2013). Polymerase chain reaction (PCR) provides a highly sensitive and effective means of detecting *T. evansi* in current, chronic, and pre-symptomatic infections. PCR assays based on amplification of *T. evansi* Type A genes, such as RoTat1.2 VSG gene (Urakawa et al. 2004), ribosomal DNA (rDNA) (Ijaz et al. 1998), r-RNA internal transcribed spacer 1 (ITS-1) (Taylor and McCormick 2008), invariant surface glycoprotein ISG-75 (Rudramurthy et al. 2013), and the VSG JN 2118Hu gene for *T. evansi* Type B (Njiru et al. 2006) have been investigated, and are, therefore, highly applicable for the confirmation of active parasitemia, given that parasite DNA is eliminated from the circulation within two days of parasite death. Recombinase polymerase amplification (RPA) (Li et al. 2020), is an easy, affordable, isothermal nucleic acid amplification technique. Its compatibility with post-PCR analysis (gel electrophoresis) makes it suitable for resource-limited laboratories and field use (Piepenburg et al. 2006; Njiru et al. 2010; Wang et al. 2017). Furthermore, the RPA lateral flow assay demonstrates high specificity for *T. evansi*, with the added utility of diagnosing active Type A infections (Li et al. 2020). Random amplified polymorphic DNA assay has been employed in the diagnosis of *T. evansi* strains (Duarte et al. 2014). Restriction fragment length polymorphism (RFLP) and loop-mediated isothermal amplification in parasite detection have also been demonstrated to be useful in trypanosome detection, especially at low parasitaemia in humans (Wastling et al. 2010).

ELISA-PCR has been employed in the detection of *T. brucei* and *T. vivax* infections in livestock (Masake et al. 2002). Additionally, multiplex-PCR has been utilised to detect *T. evansi* alongside three other haemopathogens of animals (Ganguly et al. 2020). ELISA, phase-contrast buffy coat technique, immunohistochemistry assay, indirect fluorescent assay and monoclonal antibody-based latex agglutination test are employed in the detection and diagnosis of Trypanosomosis (Nair et al. 2011; Kundu et al. 2013). Sero-diagnosis of surra exploiting recombinant VSG antigen-based ELISA, which can detect *T. evansi* in carrier cattle, has been documented (Sengupta et al. 2014). Recently, an ultraviolet-visible-near-infrared absorbance optical biosensor has been developed for the detection of *T. evansi* in blood (Theint et al. 2019).

### Prevention and control strategies

The effective management and prevention of trypanosomosis depend on the availability of rapid, reliable, and highly sensitive diagnostic tests, which are also essential for monitoring the efficacy of therapeutic and prophylactic interventions. Prevention and control measures vary with geographical location and include maintaining trypanosome-free herds through cattle import and movement control, administering trypanocidal drugs, using fly repellents, and vaccination. Surra is currently controlled through chemoprophylaxis and chemotherapy using trypanocidal agents such as diminazene aceturate, suramin, and imidocarb dipropionate. However, frequent instances of drug resistance have arisen due to the misuse and overuse of these medications (reviewed in Assefa and Shibeshi 2018), and the rise of *Trypanosoma* strains which are resistant to common trypanocidal drugs highlights an urgent need to develop effective vaccines (Mekonnen et al. 2018). These drugs are associated with adverse effects and may result in the accumulation of metabolic byproducts that contribute to hepatic and renal necrosis. Fly control is expensive and labour-intensive, and environmental pollution and climate change have complicated this issue. Biological control with sterile insects and pasture rotation could help reduce the vector population. The sterile insect technique (SIT) effectively eradicated tsetse flies from an Island (Unguja), in Zanzibar, Tanzania, by mass-releasing sterile males that mated with wild females. This led to a population decline and eventual elimination of the flies, significantly lowering trypanosomosis transmission and aiding local disease control and livestock protection efforts (Vreysen et al. 2000).

Vaccination has been an effective way to control haemopathogens, but systematic antigenic variation of the highly immunogenic VSG coat by the protozoa has greatly hindered their value as excellent vaccine candidates and therefore limited the prospects to produce a protective vaccine against trypanosomosis (Li et al. 2007). Therefore, there is no global vaccine against trypanosomosis. However, several studies have been done to identify possible vaccine candidates for inclusion in a subunit or DNA trypanosome vaccine. Thus, it is believed that soon, a DNA vaccine could be developed for the control of African trypanosomosis. Work on the development of a DNA vaccine encoding trans-sialidase (an enzyme partly involved in the pathogenesis of anaemia in trypanosomosis) in *T. brucei* has been explored (Silva et al. 2009). Recently, Gupta et al. (2019) demonstrated that DNA vaccination in mice induces both innate and T helper 1 (Th1) immune responses against Trypanosoma cruzi, the causative agent of Chagas disease. The study found that the administration of the DNA vaccine led to the activation of host immune mechanisms, specifically enhancing Th1-mediated immunity. Importantly, the study reported that, following vaccination, neither tissue parasites nor the pathology typically associated with acute trypanosomosis were detected in the vaccinated mice. This suggests that the DNA vaccine provided effective protection by preventing both parasite proliferation in tissues and the development of disease-related tissue damage during the acute phase of infection. Tewari et al. (2015) demonstrated the immunogenic properties of beta-tubulin of *T. evansi* following *T. evansi* challenge in mice. This vaccine candidate induced a specific humoral immune response of predominantly IgGa isotype and a Th-1 immune response, shown by the abundant level of Interferon-gamma. The recombinant Beta-tubulin provided protective immunity, evidenced by extended survival and better control of the parasitaemia in the immunized mice. Currently, the immunoprotective potential of recombinant paraflagellar rod (PFR) proteins of *T. evansi* was evaluated in mice, and was characterized by a balanced cytokine response, specific humoral responses in mice, including IgG1, IgG2a and IgG2b (Maharana et al. 2020). Also, these purified paraflagellar rod proteins (rTePFR1, rTePFR2) are prominent non-variable vaccine candidates for *T. evansi*, which extended the survival of the challenged mice and improved the control of parasitaemia (Maharana et al. 2020). In addition, paraflagellar rod proteins are well-conserved across various kinetoplastid parasites, making them promising vaccine candidates that could target several *Trypanosoma* species. The control of *T. evansi* infection also involves effective treatment with appropriate anti-trypanocidal drugs. Treatment of camels with melarsamine twice a month at monthly intervals for six years led to successful control and eventual eradication of *T. evansi* in a camel farm in Spain (Gutiérrez et al. 2014). For successful control of *T. evansi* in endemic areas, the role of wild animals in the epidemiology of surra needs further investigation.

## Conclusion

Blood pathogens (*Anaplasma*, *Theileria*, haemotropic *Mycoplasma* and *Trypanosoma*) of cattle are primarily associated with extravascular or intravascular haemolytic anaemia. Furthermore, co-infection of cattle with blood pathogens is a common observation in most endemic parts of the world, and laboratory diagnosis of the disease is largely based on microscopic examination of peripheral blood smears and molecular detection of the blood pathogen. Proper identification of the blood pathogens depends on factors such as the quality of the blood smear, the level of parasitaemia, technical expertise and staining technique. Diagnosing asymptomatic carrier animals is always difficult, as the level of the pathogen remains far below the detection limit of the microscope. Hence, the choice of molecular techniques for accurate detection and diagnosis of blood-borne pathogen diseases. Furthermore, immunosuppression encourages blood pathogen infection, survival and persistence in the host, and temporal host viability. Moreover, understanding the genetic architecture and immune function of the Bovine Leukocyte Antigen MHC class II and their role in immunity against blood pathogens will help to avert or reduce the use of drugs for treatment and hence, impede drug-resistant genes.

## Data Availability

Not applicable.
